# Savings Associated With Bundled Payments for Outpatient Spine Surgery Among Medicare Beneficiaries

**DOI:** 10.1001/jamahealthforum.2025.1907

**Published:** 2025-07-11

**Authors:** Austin S. Kilaru, Grace Y. Ng, Erkuan Wang, Erin Huang, Aidan P. Crowley, Jingsan Zhu, Joshua M. Liao, Said Ibrahim, Torrey Shirk, Deborah S. Cousins, Neil R. Malhotra, Amol S. Navathe

**Affiliations:** 1 Department of Neurosurgery, Massachusetts General Hospital, Harvard Medical School, Boston; 2 Department of Emergency Medicine, Perelman School of Medicine, University of Pennsylvania, Philadelphia; 3 The Parity Center, Perelman School of Medicine, University of Pennsylvania, Philadelphia; 4 Leonard Davis Institute of Health Economics, University of Pennsylvania, Philadelphia; 5 Department of Health Care Management and Economics, The Wharton School, University of Pennsylvania, Philadelphia; 6 Department of Medicine, University of Texas Southwestern Medical Center, Dallas; 7 Sidney Kimmel Medical College, Thomas Jefferson University, Philadelphia, Pennsylvania; 8 Department of Neurosurgery, Perelman School of Medicine, University of Pennsylvania, Philadelphia; 9 Corporal Michael J. Crescenz Veterans Affairs Medical Center, Philadelphia, Pennsylvania

## Abstract

**Importance:**

Few value-based payment programs have targeted outpatient surgery, although these procedures comprise most surgeries performed in hospitals. In 2018, the Centers for Medicare and Medicaid Services introduced Bundled Payments for Care Improvement Advanced (BPCI Advanced), the first episode-based payment model to include an outpatient surgical condition—spine surgery. It is not known whether bundled payments reduce spending or improve quality for outpatient surgery, despite plans to expand outpatient episodes in future models.

**Objective:**

To determine whether hospital participation in the first year of BPCI Advanced for outpatient and inpatient spine surgery (back and neck except spinal fusion procedures [BNESF]) was associated with changes in spending and quality.

**Design, Setting, and Participants:**

A retrospective cohort study using Medicare claims and differences-in-differences analysis adjusting for patient and market characteristics was conducted comparing outcomes for patients receiving outpatient and inpatient BNESF from hospitals that participated in BPCI Advanced vs those receiving these procedures from a matched comparison group of nonparticipating hospitals. Medicare beneficiaries receiving outpatient and inpatient BNESF between 2013 and 2019 were included. Analyses were conducted between March 2023 and February 2024.

**Exposures:**

Hospital participation in BPCI Advanced.

**Main Outcomes and Measures:**

The primary outcome was total episode spending, including spending incurred for the index procedure and 90-day follow-up period. Secondary outcomes included 90-day return inpatient admissions, emergency department visits, and mortality.

**Results:**

Among 14 280 patients who received outpatient BNESF, hospital participation in BPCI Advanced was associated with a differential reduction in total episode spending (−$1201; 95% CI, −2184 to −219) and return inpatient admissions (−2.2 percentage points; 95% CI, −4.2 to −0.1). For outpatient procedures, the mean (SD) age was 71.8 (8.6) years; 43.9% were women, 3.9% were Black; and 3.2% were Hispanic. Among 23 440 patients who received inpatient BNESF, hospital participation in BPCI Advanced was not associated with differential changes in total episode spending or return inpatient admissions. There were no significant changes for emergency department visits or mortality for either group.

**Conclusions and Relavance:**

In this cohort study, participation in the first year of a bundled payment program for outpatient spine surgery was associated with nearly 10% lower spending. No changes in spending were observed for similar inpatient spine surgery procedures. Further evaluation of bundled payments for outpatient surgical conditions and associated changes in care delivery is needed to inform plans to include these episodes in future models.

## Key Points

**Question:**

Was early participation in the first Medicare bundled payment episode for outpatient surgery associated with reduced spending and improved quality?

**Findings:**

In this cohort study of 14 280 patients who received outpatient spine surgery, hospital participation in bundled payments was associated with $1201 lower total episode spending and 2.2 percentage points fewer inpatient return admissions compared with similar hospitals that did not participate.

**Meaning:**

Participation in bundled payments was associated with reduced spending and improved quality for outpatient spine surgery, although longer-term evaluation of this and other outpatient conditions is needed.

## Teaser

This cohort study examines whether hospital participation in the first year of the Medicare Bundled Payments for Care Improvement Advanced program for outpatient back and neck except spinal fusion procedures was associated with reduced total spending and improved quality across an episode of care.

## Introduction

Most surgeries performed in US hospitals are outpatient procedures, in which patients are discharged shortly after the procedure or following a brief hospitalization.^1^ Many factors have driven the growth of outpatient surgery, including advancements in surgical and anesthesia techniques, patient preferences to recover at home, and payment policies.^2,3,4,5,6^ The shift from inpatient to outpatient surgery has increased value by reducing overall spending on procedures.^7^ However, total payments for outpatient surgery have substantially increased over time and improvement in quality measurement has lagged.^8,9,10,11^ Recently developed quality measures have identified variation in patient outcomes following outpatient surgery, including subsequent unplanned hospital admissions.^12^

To date, few value-based payment programs have included outpatient surgery.^11,13,14^ For inpatient surgery, episode-based payment models have generally reduced spending and maintained patient outcomes.^15,16,17,18,19,20^ Commonly known as bundled payments, these programs set target prices for all acute and postacute services related to a procedure or hospitalization, allowing clinicians and health care organizations to assume both upside and downside risk for spending as well as quality.^21,22^ Findings from Bundled Payments for Care Improvement Advanced (BPCI Advanced), the most recent model from the Centers for Medicare and Medicaid Services (CMS), demonstrated modest reductions in spending aggregated across all disease conditions.^23,24,25,26,27^

Starting in 2018, the BPCI Advanced program was the first CMS program to offer bundled payments for outpatient surgery, through an episode for outpatient back and neck except spinal fusion procedures (BNESF).^26^ The inclusion of this set of surgeries, which include lumbar laminectomy, lumbar discectomy, and anterior cervical decompression, not only permits evaluation of a new type of bundled payment episode but also exemplifies procedures that have shifted from the inpatient to outpatient setting. Spending for ambulatory spine procedures increased by 120% between 2008 and 2014.^7,28,29,30,31^ Although outpatient spine surgery has low rates of major complications, high spending and variable quality for these procedures suggest that there is potential for bundled payments to improve value.^32,33,34,35,36,37,38^

It is unclear whether bundled payments can improve value in episodes for outpatient surgeries, and prior independent evaluations of BPCI Advanced have not examined these episodes. Savings associated with bundled payments for inpatient conditions have been attributed to reduced spending on institutional postacute care, which patients typically do not receive after outpatient procedures.^15,39^ CMS recently announced plans to launch a new bundled payment model, the Transforming Episode Accountability Model (TEAM), which will include outpatient surgery episodes starting in 2026.^40^ Given that TEAM will require mandatory participation by hospitals in randomly selected US markets, there is an urgent need to examine how bundled payments for outpatient care impact spending, patient outcomes, and care delivery.

The objective of this study was to evaluate whether hospital participation in the first year of the BPCI Advanced program for outpatient BNESF was associated with reduced total spending and improved quality across an episode of care. Because surgeons may exercise discretion in determining whether to perform these procedures in the inpatient vs outpatient setting, we also examined inpatient BNESF outcomes and differential changes in patient characteristics that could indicate preferential patient selection due to model participation.

## Methods

We conducted a retrospective cohort study to compare outcomes for Medicare beneficiaries who received either outpatient or inpatient BNESF at hospitals participating in BPCI Advanced to outcomes for those who received surgery from a matched cohort of nonparticipating hospitals. The institutional review board at the University of Pennsylvania approved this study with a waiver of informed consent because the data were deidentified. This study followed the Strengthening the Reporting of Observational Studies in Epidemiology (STROBE) reporting guidelines.^41^

### Data Source and Study Population

We used a 100% sample of Medicare claims data to identify fee-for-service beneficiaries who received BNESF procedures from January 2013 to September 2019. These procedures included cervical, thoracic, and lumbar decompression of 1 or more levels without fusion (eTable 1 in Supplement 1). We used Medicare outpatient hospital claims to include procedures qualifying for 90-day BPCI Advanced outpatient episodes. We used Medicare inpatient claims to identify inpatient BNESF episodes for qualifying Medicare Severity-Diagnosis Related Groups (MS-DRGs) 518, 519, and 520.^42^ Patient enrollment and demographics were drawn from the Medicare Master Beneficiary Summary File.^43^

In accordance with BPCI Advanced program rules, we excluded beneficiaries who were not continuously enrolled in Medicare Part A and Part B, had Medicare Advantage claims, or had a primary payer other than Medicare for 180 days preceding and 90 days following the index hospitalization, and had end-stage kidney disease (eTable 2 in Supplement 1). Patients were excluded if they died, left against medical advice, or had index length of stay greater than 60 days. We obtained hospital characteristics using the Medicare Provider of Services file and the American Hospital Association Annual Survey.^44,45^ We defined markets using Hospital Referral Regions (HRRs) and obtained market characteristics from MedPAR and ACO Research-Identifiable Files.^46,47,48^

### Study Groups and Periods

We used the Medicare BPCI Advanced enrollment file to identify hospitals that participated for BNESF during model year 1 (2018 quarter 4) and model year 2 (2019). We defined separate but mutually nonexclusive groups for outpatient episode participants and inpatient episode participants (eTable 3 in Supplement 1). We defined nonparticipant hospitals as those who had qualifying BNESF episodes and did not participate in BPCI Advanced for outpatient or inpatient BNESF during model years 1 and 2. For all hospitals, we defined the baseline period as January 2013 to September 2018. The intervention period spanned October 2018 to September 2019. Data through December 2019 were included to assess 90-day postdischarge outcomes.

### Episode Construction

Per BPCI Advanced program rules, we constructed episodes spanning 90 days from the date of the procedure for outpatient beneficiaries. For inpatient beneficiaries, we constructed episodes from the day before index admission through 90 days from the date of discharge. All services reimbursed by Medicare were included in the episodes, with exclusions per program rules.^49^ We excluded BNESF episodes initiated within 90 days of any other episode for conditions included in BPCI Advanced. For patients with multiple BNESF episodes, we included either the first inpatient or outpatient episode only.

### Variables

The primary exposure was hospital BPCI Advanced participation status. We included additional covariates based on prior studies, including patient demographics (age, sex, race, ethnicity), baseline morbidity (Elixhauser comorbidities), dual eligibility for Medicare and Medicaid, disabled status, and frequency of prior hospital or postacute care use.^17,49,50^ We also included time-varying market characteristics, including size (number of Medicare fee-for-service beneficiaries), hospital Herfindahl-Hirschman index, Medicare Advantage penetration, accountable care organization (ACO) penetration, and total volume of BNESF procedures.

### Outcomes

The primary outcome was risk-standardized total episode spending, reported in 2019 dollars. For a set of secondary outcomes, we created subcategories of total episode spending, including total inpatient, total outpatient, professional services, skilled nursing facility, and home health.

We examined secondary quality outcomes for the 90-day period following discharge from the index hospitalization or procedure, including inpatient return admissions, total emergency department (ED) visits, ED visits leading to hospital admission, and mortality.^51^ For outpatient procedures, we examined the CMS quality measure for hospital outpatient surgery, 7-day unplanned hospital visits.^52^ We also examined secondary outcomes for health care use following the index hospitalization or procedure. These included discharge to postacute care, discharge to home health, skilled nursing facility (SNF) length of stay, and attendance for an outpatient clinic visit within 14 days. We examined postacute care outcomes for both inpatient and outpatient episodes although few outpatients were expected to require these services.^53^

### Statistical Analysis

We conducted separate analyses for outpatient and inpatient episodes. Following prior literature, to identify a similar comparison group to hospitals that participated in BPCI Advanced for BNESF, we used propensity score matching.^54^ We matched at the hospital level, to emulate a trial in which patients would have been randomized to the intervention based on hospital clusters. We used multivariable logistic regression to estimate propensity scores for model participation using baseline hospital and market characteristics from 2017. We used 1:3 optimal matching without replacement to identify a group of comparison hospitals at which beneficiaries receiving BNESF would comprise the comparison group. We compared standardized mean differences for hospital and market characteristics used to estimate propensity scores as well as the overlap in propensity score distributions before and after matching (eTables 4 and 5 in Supplement 1). We accounted for individual covariates with residual imbalances (standardized mean difference, <0.25) in the postmatching regression model.^55,56^

We used a differences-in-differences method to examine changes in outcomes before and after BPCI Advanced participation. First, we examined parallel trends for all outcomes in the baseline period for participant vs matched nonparticipant hospitals (eTable 6 in Supplement 1). Then, we used multivariable generalized linear models to estimate differential changes in outcomes (eTable 7 in Supplement 1). We obtained the differences-in-differences estimate by interacting BPCI Advanced participation and postparticipation time indicators. All models included patient characteristics and time-varying market characteristics. Models included time (quarter) and market (beneficiary hospital referral region) fixed effects as well as MS-DRG fixed effects for the inpatient cohort. All models used robust standard errors clustered at the hospital level. Statistical tests were 2-tailed and considered significant at α = .05. We plotted risk-standardized trends for the primary outcome by using the marginal standardization approach.^51^ Analyses were performed using SAS Enterprise Guide (version 7.15; SAS Institute, Inc). Analyses were conducted between March 2023 and February 2024.

### Sensitivity Analyses

We conducted sensitivity analyses to examine the robustness of the main analysis. First, we used hospital, rather than market, fixed effects to account for any residual imbalances in time-invariant hospital characteristics after matching.^55^ Second, we repeated the main analyses using a 1:1 matching approach. Third, we repeated the main analyses without performing matching, including all eligible hospitals in a multivariable generalized linear model with an indicator for BPCI-A participation and hospital fixed effects, as well as adjusting for all patient, hospital, and market characteristics. Finally, we repeated the main analysis using a shorter preintervention period, starting in 2016, to account for shifts in volumes of outpatient and inpatient procedures occurring in the preceding years.

We assessed for evidence of patient selection that may have occurred after participation in the model. We used differences-in-differences models to estimate differential changes in patient characteristics before and participation in BPCI Advanced. We also examined whether the volume of either inpatient or outpatient surgeries changed before and after participation in BPCI Advanced, separately examining hospitals in groups based on participation status (both inpatient and outpatient, outpatient only, inpatient only).

## Results

### Study Population

The study included 14 280 patients who received outpatient BNESF between January 2013 and September 2019. Of these, 3748 had BNESF at 19 hospitals participating in BPCI Advanced, and 10 532 had BNESF at the matched group of 57 nonparticipant hospitals. For outpatient procedures, the mean (SD) age was 71.8 (8.6) years; 43.9% were women, 3.9% were Black; and 3.2% were Hispanic (Table 1).

**Table 1.** Patient Characteristics for Outpatient Back and Neck Except Spinal Fusion Episodes, Before (January 2013-September 2018) and After (October 2018-September 2019) BPCI-A Participation

Characteristic	No. (%)			
	Patients receiving care from participating hospitals		Patients receiving care from matched nonparticipating hospitals	
	Before (n = 3155)	After (n = 593)	Before (n = 8731)	After (n = 1801)
Age, mean (SD), y	72.0 (8.5)	73.1 (8.0)	71.5 (8.8)	72.3 (8.4)
Sex				
Female	1382 (43.8)	245 (41.3)	3864 (44.3)	782 (43.4)
Male	1773 (56.2)	348 (58.7)	4867 (55.7)	1019 (56.5)
Race and ethnicity				
Black	173 (5.5)	30 (5.1)	317 (3.6)	42 (2.3)
Hispanic	143 (4.5)	25 (4.2)	233 (2.7)	54 (3.0)
White	2645 (83.8)	482 (81.3)	7779 (89.1)	1587 (88.1)
Other^a^	194 (6.2)	56 (9.4)	402 (4.6)	118 (6.6)
Dual eligible	260 (8.2)	56 (9.4)	806 (9.2)	125 (6.9)
Disabled	297 (9.4)	41 (6.9)	995 (11.4)	158 (8.8)
Total No. of Elixhauser comorbidities, mean (SD)	3.8 (2.5)	3.6 (2.4)	3.7 (2.5)	3.6 (2.4)
Most common Elixhauser comorbidities				
Hypertension	2545 (80.7)	450 (75.6)	6848 (78.4)	1391 (77.2)
Diabetes	990 (31.4)	167 (28.2)	2625 (30.1)	541 (30.0)
Chronic lung disease	786 (24.9)	166 (28.0)	2216 (25.4)	446 (24.8)
Hypothyroidism	718 (22.8)	131 (22.1)	1973 (22.6)	379 (21.0)
Obesity	686 (21.7)	140 (23.6)	1921 (22.0)	429 (23.8)
Prior hospitalization in the past 1 y	444 (14.1)	62 (10.5)	1150 (13.2)	224 (12.4)
Prior skilled nursing facility or inpatient rehabilitation use in preceding 1 y	60 (1.9)	7 (1.2)	164 (1.9)	30 (1.7)

Abbreviation: BPCI-A, Bundled Payments for Care Improvement Advanced.

^a^ Includes Asian, North American Native, and unknown race and ethnicity.

The study also included 23 440 patients who received inpatient BNESF during the same period. There were 6764 patients who had BNESF at 41 hospitals participating in BPCI Advanced, and 16 676 had BNESF at the matched group of 123 nonparticipant hospitals (Table 2). Twelve hospitals participated in both inpatient and outpatient BNESF episodes. Hospital and market characteristics are included in eTables 4 and 8 in Supplement 1.

**Table 2.** Patient Characteristics for Inpatient Back and Neck Except Spinal Fusion Episodes, Before (January 2013 to September 2018) and After (October 2018 to September 2019) BPCI-A Participation

Characteristic	No. (%)			
	Patients receiving care from participating hospitals		Patients receiving care from matched nonparticipating hospitals	
	Before (n = 6142)	After (n = 622)	Before (n = 15 010)	After (n = 1666)
Age, mean (SD), y	73.1 (9.3)	73.2 (9.1)	72.8 (9.7)	73 (9.4)
Sex				
Female	2817 (45.9)	309 (49.7)	6730 (44.8)	774 (46.5)
Male	3325 (54.1)	313 (50.3)	8280 (55.2)	892 (53.5)
Race and ethnicity				
Black	355 (5.8)	42 (6.8)	632 (4.2)	88 (5.3)
Hispanic	158 (2.6)	24 (3.9)	455 (3.0)	63 (3.8)
White	5357 (87.2)	530 (85.2)	13262 (88.4)	1414 (84.9)
Other^a^	272 (4.4)	26 (4.2)	661 (4.4)	101 (6.1)
Dual eligible	630 (10.3)	77 (12.4)	1875 (12.5)	218 (13.1)
Disabled	664 (10.8)	70 (11.3)	1789 (11.9)	202 (12.1)
Total No. of Elixhauser comorbidities, mean (SD)	4.7 (2.8)	4.8 (3.0)	4.7 (2.8)	4.5 (2.7)
Most common Elixhauser comorbidities				
Hypertension	5322 (86.6)	520 (83.6)	12837 (85.5)	1393 (83.6)
Diabetes	2356 (38.4)	246 (39.5)	5556 (37.0)	599 (36.0)
Chronic lung disease	1874 (30.5)	185 (29.7)	4572 (30.5)	470 (28.2)
Hypothyroidism	1561 (25.4)	154 (24.8)	3842 (25.6)	400 (24.0)
Obesity	1630 (26.5)	194 (31.2)	3835 (25.5)	489 (29.4)
Prior hospitalization in the past 1 y	1278 (20.8)	147 (23.6)	3125 (20.8)	333 (20.0)
Prior skilled nursing facility or inpatient rehabilitation use in preceding 1 y	391 (6.4)	46 (7.4)	992 (6.6)	116 (7.0)

Abbreviation: BPCI-A, Bundled Payments for Care Improvement Advanced.

^a^ Includes Asian, North American Native, and unknown race and ethnicity.

### Outpatient Episodes

For patients receiving outpatient BNESF at hospitals participating in BPCI Advanced, unadjusted total episode spending decreased between baseline and intervention periods from $13 100 to $13 086, whereas spending increased from $11 677 to $12 946 for patients at nonparticipant hospitals (Table 3). In adjusted analyses, participation in BPCI Advanced was associated with a reduction in spending of −$1201 (95% CI, −$2184 to −$219). Plots of risk-standardized trends illustrated the decrease in spending among participant hospitals relative to nonparticipants (Figure). We examined subcategories of total episode spending (eTable 9 in Supplement 1). For outpatient episodes, participation in BPCI Advanced was associated with decreased spending for professional services (−$245; 95% CI, −$466 to −$24) and inpatient spending (−$675; 95% CI, −$1133 to −$217). There were no significant changes for other subcategories.

**Table 3.** Differential Changes in Spending, Quality, and Utilization by Hospital Participation in BPCI-A Outpatient BNESF Episodes

Variable	Participating hospitals, %		Matched nonparticipating hospitals, %		Adjusted differences-in-differences estimate (95% CI)
	Before (n = 3155)	After (n = 593)	Before (n = 8731)	After (n = 1801)	
Spending					
Total episode spending, $^a^	13 100	13 086	11 677	12 946	−1201 (−2184 to −219)
Quality					
90-d Inpatient return admission	8.1	6.7	6.9	7.9	−2.2 (−4.2 to −0.1)
90-d ED visit	17.7	16.5	16.7	14.5	0.4 (−3.3 to 4.1)
90-d ED visit leading to inpatient admission	5.4	4.9	4.4	4.8	−0.5 (−2.1 to 1.1)
90-d Mortality	0.3	0.2	0.2	0.2	0 (−0.5 to 0.4)
7-d Unplanned hospital visit^b^	9.7	6.6	8.3	7.9	−2.6 (−6.1 to 0.8)
Utilization					
Discharge to post-acute care facility^c^	0.7	0.5	0.3	0.3	−0.1 (−0.7 to 0.5)
Skilled nursing facility length of stay, d	0.5	0.6	0.3	0.3	0.2 (−0.2 to 0.7)
Discharge to home health	0.6	0.8	0.5	0.3	0 (−1.4 to 1.4)
14-d Clinic visit	26.5	29.0	27.9	27.1	3.9 (−1.8 to 9.6)

Abbreviations: BPCI-A, Bundled Payments for Care Improvement Advanced; BNESF, back and neck except spinal fusion procedures; ED, emergency department.

^a^ Includes index hospitalization (admission or outpatient stay) as well as 90 days after discharge.

^b^ Includes ED visits, observation visits, and hospital admissions within 7 days.

^c^ Includes skilled nursing facility, inpatient rehabilitation facility, and long-term acute care.

**Figure.**
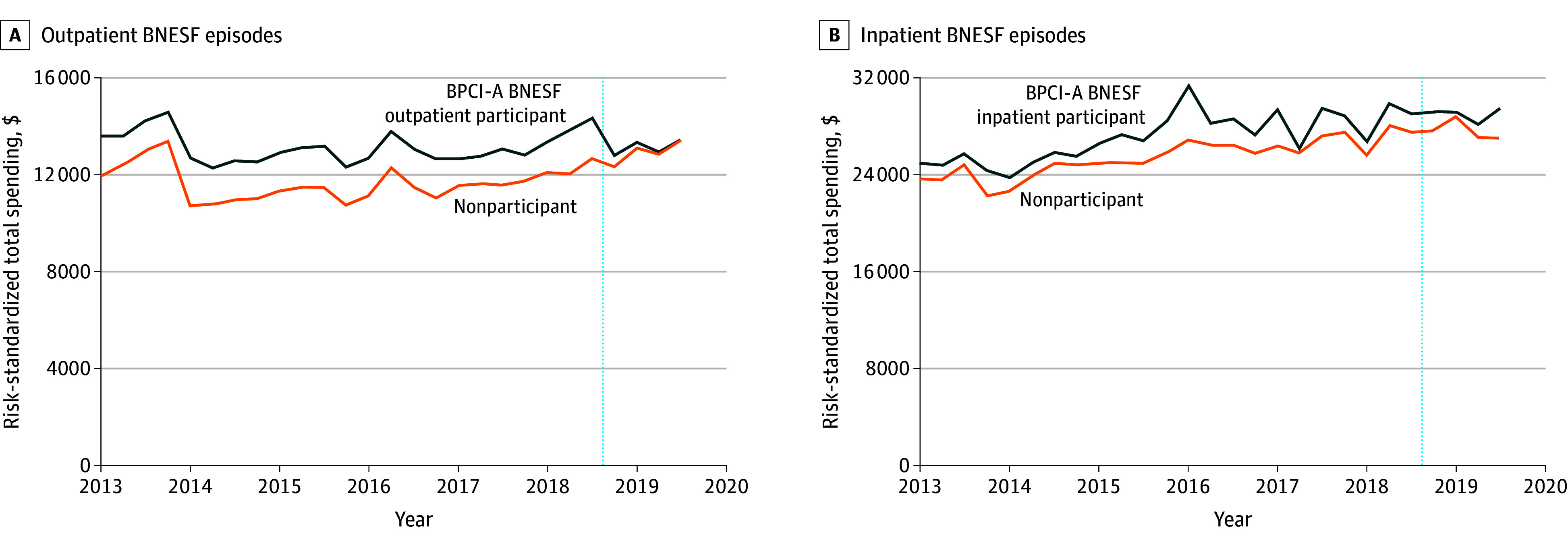
Risk-Standardized Total Spending Bundled Payments for Care Improvement Advanced (BPCI-A) back and neck except spinal fusion procedures (BNESF) participants and matched nonparticipants, by year. The vertical dotted blue line indicates the start of the BPCI-A model.

Patients who received outpatient BNESF at participating hospitals had fewer 90-day inpatient return admissions compared with patients at nonparticipating hospitals (−2.2 percentage points; 95% CI, −4.2 to −0.1) (Table 3). However, there was no significant change in 90-day return admissions originating from the ED (−0.5 percentage points; 95% CI, −2.1 to 1.1). We further describe categories of inpatient return admissions for participant and nonparticipant hospitals in eTable 10 in Supplement 1. Patients at participating hospitals did not have significantly fewer 7-day unplanned hospital visits (−2.5 percentage points; 95% CI, −5.9 to 1.0). There were no significant changes in the rate of ED visits, rate of ED visits leading to inpatient admission, or mortality at 90 days, and there were no significant changes in use of postacute or home health care.

### Inpatient Episodes

For inpatient BNESF, total episode spending increased for hospitals participating in BPCI Advanced between baseline and intervention periods from $26 581 to $29 019 (Table 4). Spending also increased from $25 075 to $27 678 at nonparticipant hospitals. In adjusted analyses, participation in inpatient BPCI Advanced was not associated with a change in total spending (−$217; 95% CI, −$2334 to $1900). There were no significant changes in quality or utilization outcomes including use of postacute care, apart from an increase in ED visits leading to inpatient admission (4.0 percentage points; 95% CI, 0.5 to 3.5).

**Table 4.** Differential Changes in Spending, Quality, and Utilization by Hospital Participation in BPCI-A Inpatient BNESF Episodes

Variable	Participating hospitals, %		Matched nonparticipating hospitals, %		Adjusted differences-in-differences estimate (95% CI)
	Before (n = 6142)	After (n = 622)	Before (n = 15010)	After (n = 1666)	
Total episode spending, $^a^	26 581	29 019	25 075	27 678	−217 (−2334 to 1900)
Quality					
90-d Inpatient return admission	14.0	17.2	13.7	13.3	3.4 (−0.4 to 7.1)
90-d ED visit	23.4	28.0	24.0	25.0	3.3 (−1.4 to 8.0)
90-d ED visit leading to inpatient admission	11.0	15.1	10.2	9.9	4.0 (0.5 to 7.5)
90-d Mortality	1.3	1.9	1.3	2.0	0.2 (−1.3 to 1.6)
Utilization					
Discharge to postacute care facility^b^	31.3	32.6	27.7	32.5	−3.9 (−8.8 to 1.0)
Skilled nursing facility length of stay, d	6.2	6.2	5.5	6.3	−1.0 (−2.4 to 0.3)
Discharge to home health	10.0	13.0	10.3	11.3	2.2 (−2.0 to 6.5)
14-d Clinic visit	34.6	34.7	31.5	32.8	−0.9 (−5.9 to 4.0)

Abbreviations: BPCI-A, Bundled Payments for Care Improvement Advanced; BNESF, back and neck except spinal fusion procedures; ED, emergency department.

^a^ Includes index hospitalization (admission or outpatient stay) and 90 days after discharge.

^b^ Includes skilled nursing facility, inpatient rehabilitation facility, and long-term acute care.

### Sensitivity Analyses

For all models, results were similar to the main findings for the sensitivity analyses that used hospital fixed effects and 1:1 matching (eTable 11 in Supplement 1). For the analysis conducted without matching, the primary outcome for the outpatient cohort was no longer significant, remaining similar with regard to direction but with attenuated magnitude. For the analyses using a shorter preperiod, results for the primary outcome remained significant.

For outpatient episodes, we observed no differential changes in patient characteristics including age, race, and comorbidity burden (eTable 12 in Supplement 1). We did observe that the percentage of patients dually eligible for Medicare and Medicaid increased for participating hospitals compared with nonparticipants (3.5 percentage points; 95% CI, 0.7-6.4). For inpatient episodes, we observed no differential changes in patient characteristics. Finally, we found no evidence that the volume of either inpatient or outpatient procedures changed from before and after the intervention, regardless of whether hospitals participated in outpatient episodes, inpatient episodes, or both (eTable 13 in Supplement 1).

## Discussion

In this study of the first outpatient surgical episode in a Medicare bundled payment model, we found that hospital participation during the early phase of the program was associated with decreased spending and fewer return hospital admissions for outpatient spine surgery. We did not observe similar changes for inpatient episodes, consistent with some prior studies that have examined bundled payments for spine procedures but not others.^15,27,57,58,59^ These findings, notable for the difference in episode type as well as the mechanism by which savings were generated, offer preliminary evidence for health care organizations and policymakers as they anticipate the inclusion of outpatient surgery in a national mandatory bundled payment model in 2026.^40^

Few studies have evaluated the impact of bundled payments on outpatient surgery and, more generally, any episodic care delivered in the outpatient setting. One prior study^14^ examined a Medicare Advantage bundled payment program that allowed physician groups to select an outpatient hospital setting for lower extremity joint replacement. Although the program demonstrated reduced spending, savings were generated by shifting inpatient procedures to outpatient status. That program design differed significantly from BPCI Advanced, which treated outpatient and inpatient surgeries as separate episode types. Furthermore, although bundled payments for inpatient surgery have generated savings for orthopedic procedures, those savings have been concentrated in reductions of postacute care rather than surgical complications or return hospital admissions.

In this context, our findings are novel and merit further evaluation across other conditions and over a longer time period. As expected, we observed little use of postacute care following outpatient procedures. Instead, decreased total spending was accompanied by a reduction in inpatient admissions and associated spending on inpatient and professional services. Our findings could be explained by improved quality performance. Another potential explanation is that complications were mitigated in the outpatient setting, through close monitoring and improved care coordination. Strengthened care transitions is an explicit goal for future bundled payment programs, although little evidence suggests that improved transitions have contributed to reduced spending in prior models.^60^ Of note, although we found that there were fewer inpatient return admissions in the outpatient cohort, there was no significant change in return admissions originating from the ED, suggesting that this outcome may be due to reduced elective or direct admissions.

Another potential explanation for these findings is the selection of healthier patients into outpatient episodes based on clinician discretion. To account for the findings in this study, however, patient selection would have had to differentially accelerate among hospitals participating in BPCI Advanced. Overall, we did not observe advantageous changes in observable patient characteristics. Concerns over selection have since been addressed by rule changes in this program that now require health care organizations to participate in both episode types.^26^ Other explanations for our findings might include differential spending increases among the matched comparison hospitals following the intervention or increased spending for treatment hospitals prior to the intervention with subsequent reversion to the mean. Although spending was observed to increase among the comparison hospitals following the intervention, these increases continued along the same trend that was observed for the years preceding the intervention. Furthermore, we did not observe differential changes in total episode spending or its components between treatment and comparison groups prior to the intervention.

Finally, it is worth noting the additional complexities that can arise from another form of selection, namely selective participation of hospitals in the program. An inherent feature of voluntary models is that health care organizations that participate likely have confidence in their ability to reduce spending against the defined benchmark. Although the differences-in-differences design minimized the impact of selective hospital participation on these findings, it is still notable that participating hospitals had higher baseline spending in both cohorts.

This study has important implications for policymakers as they consider the future of value-based payment. Our findings suggest that including outpatient surgery in bundled payment programs might have advantages, with the potential to generate savings independent of reductions in postacute care. Additional advantages may include mitigation of incentives to select healthier patients into inpatient episodes when there is clinical discretion for episode type. For example, including outpatient episodes might have offset the unintended effects observed in the inpatient-only Comprehensive Care for Joint Replacements program related to slower uptake of outpatient knee replacement surgery.^61^ CMS specified that the TEAM model will initially include 5 surgical conditions, 2 of which may be performed as outpatient procedures: lower extremity joint replacement and spinal fusion. However, the model remains open to expand to more outpatient procedures, both medical and surgical.^62^

Overall, our study identified savings associated with outpatient spine episodes. However, for inpatient spine surgery episodes the picture is less clear. Our findings for the inpatient cohort differ from a recent study^27^ of BPCI Advanced that compared changes in spending between medical and surgical episode types. For inpatient BNESF, the study reported a significant reduction in total episode spending for the same study period, applying broadly similar methods. Although the reason for these divergent findings is not clear and could be related to specific study design choices, it is notable that the magnitude of total episode spending reported for inpatient BNESF episodes was considerably lower than those reported in this study.

### Limitations

This cohort study had limitations. First, the study only included episodes for the first year of the program, to avoid changes due to the COVID-19 pandemic, program rule changes, and the departure of hospitals from the program.^36^ Second, this study did not include episodes initiated by physician groups, although hospitals were the predominant participant type for these episodes.^24^ Third, this study did not consider procedures performed in ambulatory surgery centers, which were not allowed to participate in BPCI Advanced.^10^ Fourth, this study may not have fully accounted for factors related to patient selection into outpatient or inpatient episodes as well as shifts to other types of care, including medical management or spinal fusion, that were unobserved in available data. However, we neither found evidence of differential changes in observed characteristics nor did we observe shifts in volumes between inpatient and outpatient procedures. Furthermore, selection at the hospital level was mitigated through the difference-in-differences design and sensitivity analysis using hospital fixed-effects. Fifth, intervention and comparison groups exhibited residual imbalances across some hospital characteristics, although our findings were unchanged when we accounted for these differences in a sensitivity analysis. Finally, this analysis did not account for incentives or penalties for hospitals through bundled payments, focusing this analysis on spending for health care services rather than the total net gain or loss for CMS.

## Conclusions

Participation in the first year of a bundled payment program for outpatient spine surgery was associated with significantly lower spending and fewer readmissions during an episode of care. No changes in spending or quality were observed for equivalent procedures performed in the inpatient setting. Additional evaluation of BNESF and other outpatient conditions in later program years is needed to confirm these findings, which will inform current plans by payers and policymakers to expand bundled payments for outpatient care.

## Notes

Supplement 1. eTable 1. Billing Code Identifiers for Back and Neck Except Spinal Fusion (BNESF) Procedures eFigure 1. Cohort Flow Chart – Outpatient Episodes eFigure 2. Cohort Flow Chart – Inpatient Episodes Table 2. Number of hospitals participating in inpatient and outpatient episodes of back and neck except spinal fusion procedures eTable 3. Characteristics for outpatient participant and non-participant hospitals, pre/post matching eTable 4. Characteristics for inpatient participant and non-participant hospitals, pre/post matching eFigure 3. Balance plots for propensity scores before and after matching – outpatient cohort eFigure 4. Balance plots for propensity scores before and after matching – inpatient cohort eFigure 5. Parallel trends for primary and selected secondary outcomes eMethods Primary model specification eTable 5. Baseline market characteristics eTable 6. Episode spending, by category, for inpatient and outpatient episodes eTable 7. Return admissions occurring after index hospitalization, by DRG categories eTable 8. Sensitivity analyses – differential changes in spending, quality, and utilization by hospital participation in BPCI-A outpatient BNESF episodes, with models that use eTable 9. Analysis for patient selection effects – difference-in-difference analysis of key patient characteristics eTable 10. Analysis for patient selection effects – analysis of shifts in volume between inpatient and outpatient surgeries

Supplement 2. Data Sharing Statement
