# Supplementary material for: Savings Associated With Bundled Payments for Outpatient Spine Surgery Among Medicare Beneficiaries
Source: JAMA Health Forum. 2025 Jul 11;6(7):e251907. doi: 10.1001/jamahealthforum.2025.1907 (PMC12254892; doi:10.1001/jamahealthforum.2025.1907)
Supplement: Supplement 1. — eTable 1. Billing Code Identifiers for Back and Neck Except Spinal Fusion (BNESF) Procedures eFigure 1. Cohort Flow Chart – Outpatient Episodes eFigure 2. Cohort Flow Chart – Inpatient Episodes Table 2. Number of hospitals participating in inpatient and outpatient episodes of back and neck except spinal fusion procedures eTable 3. Characteristics for outpatient participant and non-participant hospitals, pre/post matching eTable 4. Characteristics for inpatient participant and non-participant hospitals, pre/post matching eFigure 3. Balance plots for propensity scores before and after matching – outpatient cohort eFigure 4. Balance plots for propensity scores before and after matching – inpatient cohort eFigure 5. Parallel trends for primary and selected secondary outcomes eMethods Primary model specification eTable 5. Baseline market characteristics eTable 6. Episode spending, by category, for inpatient and outpatient episodes eTable 7. Return admissions occurring after index hospitalization, by DRG categories eTable 8. Sensitivity analyses – differential changes in spending, quality, and utilization by hospital participation in BPCI-A outpatient BNESF episodes, with models that use eTable 9. Analysis for patient selection effects – difference-in-difference analysis of key patient characteristics eTable 10. Analysis for patient selection effects – analysis of shifts in volume between inpatient and outpatient surgeries [file jamahealthforum-e251907-s001.pdf]

Supplemental Online Content

Kilaru AS, Ng GY, Wang E, et al. Savings Associated with bundled payments for outpatient spine surgery among medicare beneficiaries. *JAMA Health Forum*. 2025;6(7):e251907. doi:10.1001/jamahealthforum.2025.1907

eTable 1. Billing Code Identifiers for Back and Neck Except Spinal Fusion (BNESF) Procedures
eFigure 1. Cohort Flow Chart – Outpatient Episodes
eFigure 2. Cohort Flow Chart – Inpatient Episodes
Table 2. Number of hospitals participating in inpatient and outpatient episodes of back and neck except spinal fusion procedures
eTable 3. Characteristics for outpatient participant and non-participant hospitals, pre/post matching
eTable 4. Characteristics for inpatient participant and non-participant hospitals, pre/post matching
eFigure 3. Balance plots for propensity scores before and after matching – outpatient cohort
eFigure 4. Balance plots for propensity scores before and after matching – inpatient cohort
eFigure 5. Parallel trends for primary and selected secondary outcomes
eMethods
Primary model specification
eTable 5. Baseline market characteristics
eTable 6. Episode spending, by category, for inpatient and outpatient episodes
eTable 7. Return admissions occurring after index hospitalization, by DRG categories
eTable 8. Sensitivity analyses – differential changes in spending, quality, and utilization by hospital participation in BPCI-A outpatient BNESF episodes, with models that use
eTable 9. Analysis for patient selection effects – difference-in-difference analysis of key patient characteristics
eTable 10. Analysis for patient selection effects – analysis of shifts in volume between inpatient and outpatient surgeries

This supplemental material has been provided by the authors to give readers additional information about their work.

© 2025 Kilaru AS et al. *JAMA Health Forum*.

S1. Table

Billing Code Identifiers for Back and Neck Except Spinal Fusion (BNESF) Procedures

Billing Code	Definition
**Outpatient Procedures – Healthcare Common Procedure Coding System (HCPCS)**	
62287	Decompression procedure, percutaneous, of nucleus pulposus of intervertebral disc, any method utilizing needle-based technique to remove disc material under fluoroscopic imaging or other form of indirect visualization, with discography and/or epidural injection(s) at the treated level(s), when performed, single or multiple levels, lumbar
63005	Laminectomy with exploration and/or decompression of spinal cord and/or cauda equina, without facetectomy, foraminotomy or discectomy (e.g., spinal stenosis), 1 or 2 vertebral segments; lumbar, except for spondylolisthesis
63011	Laminectomy with exploration and/or decompression of spinal cord and/or cauda equina, without facetectomy, foraminotomy or discectomy (e.g., spinal stenosis), 1 or 2 vertebral segments; sacral
63012	Laminectomy with removal of abnormal facets and/or pars inter-articularis with decompression of cauda equina and nerve roots for spondylolisthesis, lumbar (Gill type procedure)
63017	Laminectomy with exploration and/or decompression of spinal cord and/or cauda equina, without facetectomy, foraminotomy or discectomy (e.g., spinal stenosis), more than 2 vertebral segments; lumbar
63030	Laminectomy with exploration and/or decompression of spinal cord and/or cauda equina, without facetectomy, foraminotomy or discectomy (e.g., spinal stenosis), more than 2 vertebral segments; lumbar
63040	Laminotomy (hemilaminectomy), with decompression of nerve root(s), including partial facetectomy, foraminotomy and/or excision of herniated intervertebral disc, re-exploration, single interspace; cervical
63042	Laminotomy (hemilaminectomy), with decompression of nerve root(s), including partial facetectomy, foraminotomy and/or excision of herniated intervertebral disc, re-exploration, single interspace; lumbar
63045	Laminectomy, facetectomy and foraminotomy (unilateral or bilateral with decompression of spinal cord, cauda equina and/or nerve root[s], [e.g., spinal or lateral recess stenosis]), single vertebral segment; cervical
63046	Laminectomy, facetectomy and foraminotomy (unilateral or bilateral with decompression of spinal cord, cauda equina and/or nerve root[s], [e.g., spinal or lateral recess stenosis]), single vertebral segment; thoracic
63047	Laminectomy, facetectomy and foraminotomy (unilateral or bilateral with decompression of spinal cord, cauda equina and/or nerve root[s], [e.g., spinal or lateral recess stenosis]), single vertebral segment; lumbar
63056	Transpedicular approach with decompression of spinal cord, equina and/or nerve root(s) (e.g., herniated intervertebral disc), single segment; lumbar (including transfacet, or lateral extraforaminal approach) (e.g., far lateral herniated intervertebral disc)
63075	Discectomy, anterior, with decompression of spinal cord and/or nerve root(s), including osteophytectomy; cervical, single interspace
**Inpatient Procedures – Medicare-Severity Diagnosis Related Groups (MS-DRG)**	
518 (after 10/1/2014)	Back and neck procedures except spinal fusion with MCC or disc device or neurostimulator
519 (after 10/1/2014)	Back and neck procedures except spinal fusion with CC
520 (after 10/1/2014)	Back and neck procedures except spinal fusion without CC/MCC
490 (before 10/1/2014)	Back & neck procedure except spinal fusion with CC/MCC or disc device or neurostimulator
491 (before 10/1/2014)	Back & neck procedure except spinal fusion without CC/MCC

© 2025 Kilaru AS et al. *JAMA Health Forum*.

S2. Figure

Cohort Flow Chart – Outpatient Episodes

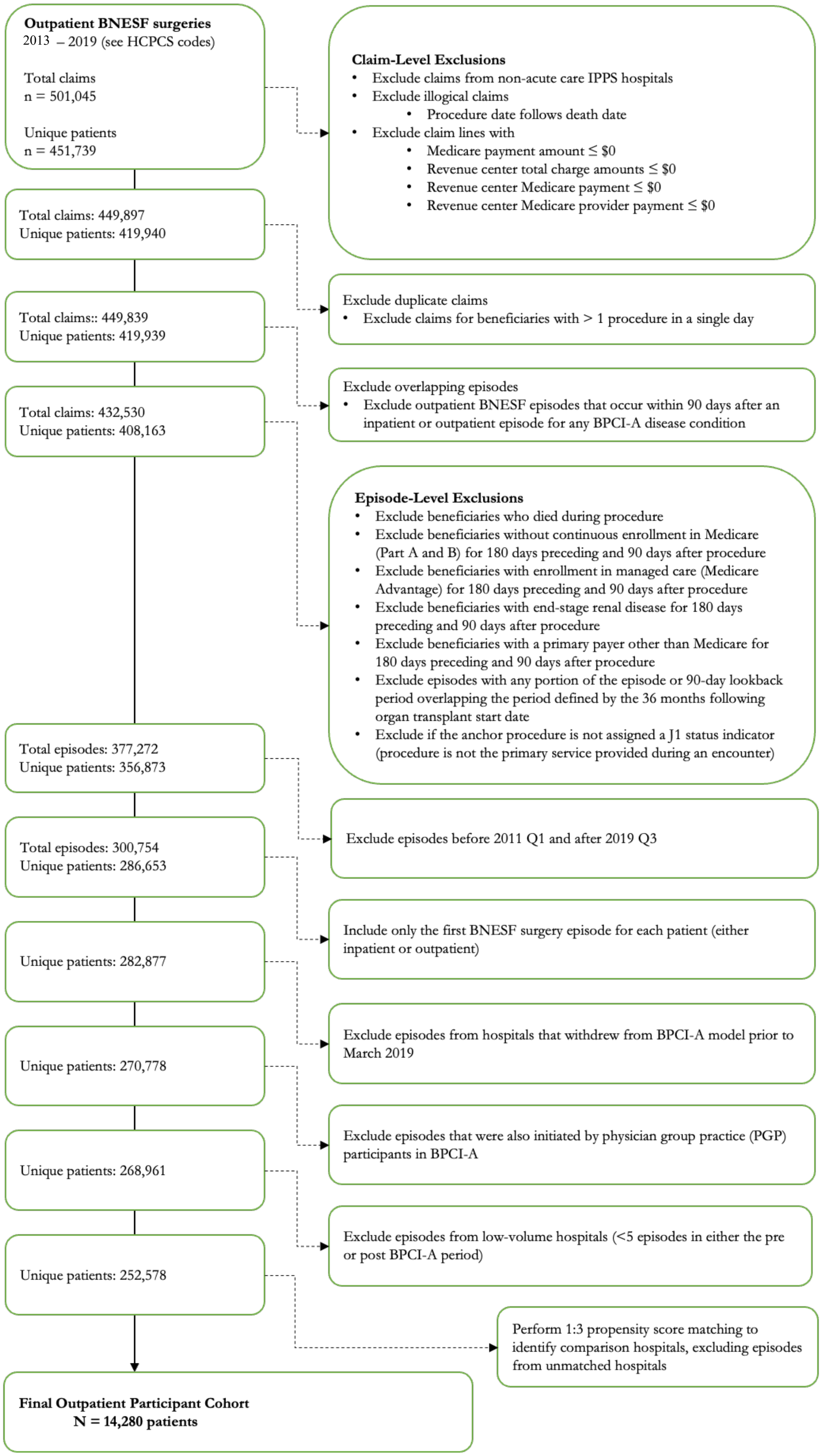

© 2025 Kilaru AS et al. *JAMA Health Forum*.

2013

Cohort Flow Chart – Inpatient Episodes

252,105

© 2025 Kilaru AS et al. *JAMA Health Forum*.

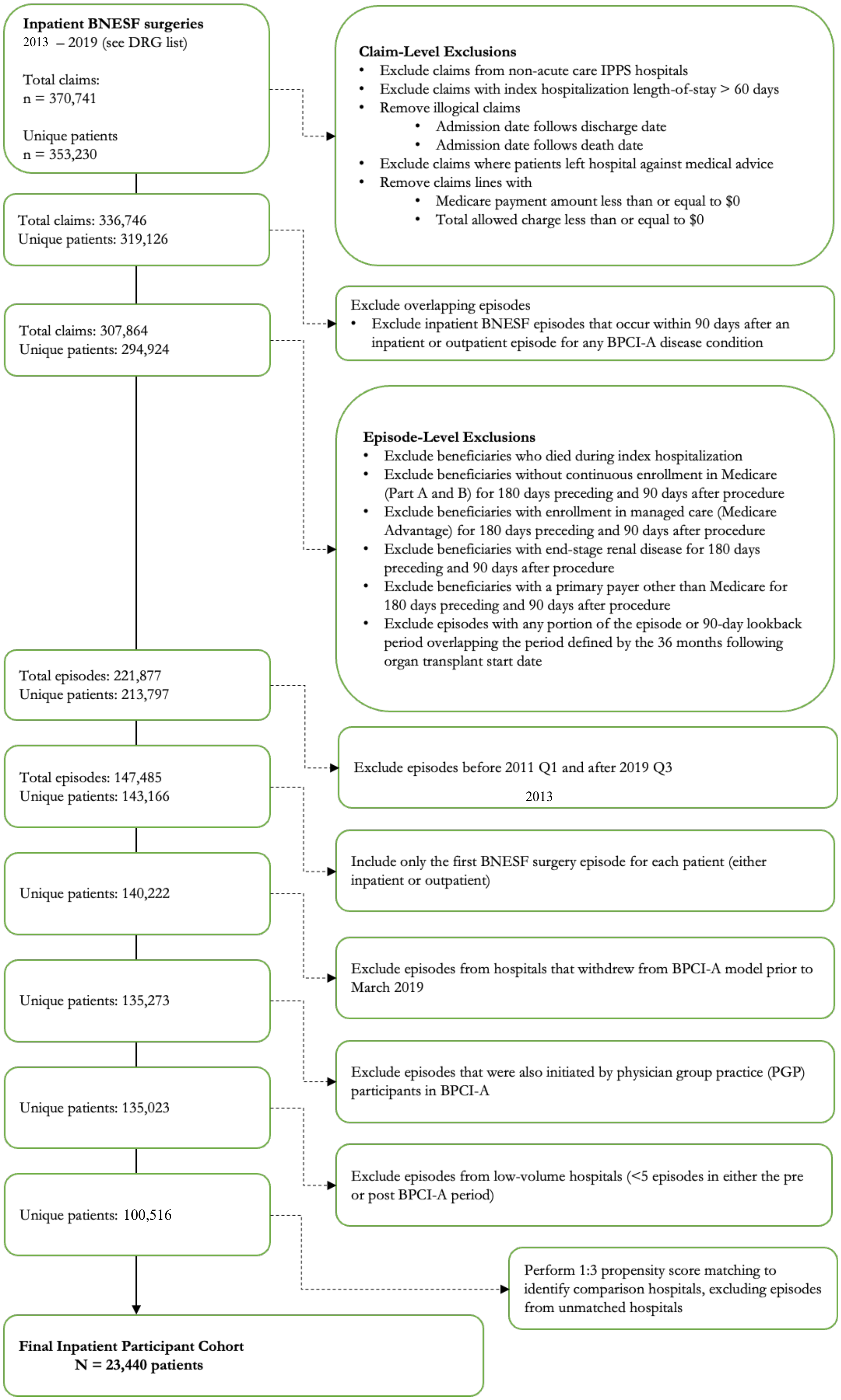

© 2025 Kilaru AS et al. *JAMA Health Forum*.

S3. Table
Number of hospitals participating in inpatient and outpatient episodes of back and neck except spinal fusion procedures

In this study, we analyzed inpatient and outpatient episode participants separately. A majority of hospitals participating in the outpatient episode (12 of 19) also participated in the equivalent inpatient episode.

**Hospital Participation in BPCI Advanced**	**n**
Inpatient BNESF episode participants	41
Outpatient BNESF episode participants	19
Inpatient non-participant hospitals in matched comparison group	123
Outpatient non-participant hospitals in matched comparison group	57
Hospitals participating in both inpatient and outpatient BNESF episodes	12

© 2025 Kilaru AS et al. *JAMA Health Forum*.

S4. Table

A. Characteristics of outpatient participant and matched non-participant hospitals, pre/post matching

	Before Matching			After Matching		
	Participants (n = 19)	Non-Participants (n = 1281)	Absolute Standard Mean Difference	Participants (n = 19)	Non-Participants (n = 57)	Absolute Standard Mean Difference
Predicted Probability of BPCI-A Participation (Propensity Score)			1.18			0.01
**Hospital-Level Characteristics**						
**Ownership**, %						
Non-Profit	84.2	69.7	0.34	84.2	75.4	0.21
For-Profit	10.5	21.7	0.31	10.5	19.3	0.24
Government	5.3	8.5		5.3	5.3	
**Teaching status**, %						
Major	26.3	13.3	0.33	26.3	29.8	0.08
Minor	68.4	56.8	0.24	68.4	56.1	0.23
None	5.3	29.9		5.3	14.0	
**Annual Medicare days**, % of total inpatient days	45.8	51.2	0.51	45.8	44.5	0.12
**Total hospital bed count**, %						
Large	36.8	40.4	0.19	36.8	31.6	0.00
Medium	52.6	43.9	0.10	52.6	56.1	0.17
Small	10.5	15.7		10.5	12.2	
**Health system affiliation,** %						
Affiliated	78.9	77.7	0.03		66.7	0.19
None	21.1	22.2			33.3	
**Urban status,** %			--			--
Urban	100.0	99.0		100.0	100.0	
Rural	0.0	1.0		0.0	0.0	
**Disproportionate share payment (2017),** mean, $	3,436,045	2,353,294	0.30	3,436,045	3,863,127	0.11

© 2025 Kilaru AS et al. *JAMA Health Forum*.

**BNESF volume (2017)**, mean, no.	22.2	19.0	0.17	22.2	24.7	0.34
**Annual hospital market share (2013 – 2018Q3)**, mean, %	11.2	16.1	0.34	11.2	9.9	0.08
**Discharges to highest volume SNF**, % of SNF discharges	22.2	24.9	0.16	22.2	23.3	0.06
**Hospital Market (Hospital Referral Region) Characteristics**						
**Population**, mean, no.	2,580,870	2,047,609	0.26	2,580,870	2,816,979	0.21
**Low-income status**, %	31.9	47.7	0.73	31.9	32.6	0.03
**SNF beds**, per 10000 Medicare beneficiaries	0.06	0.06	0.16	0.06	0.06	0.07
**Hospital Herfindahl-Hirschman Index**, score	1124	1950	0.65	1124	974	0.11
BNESF, back and neck procedure except spinal fusion; SNF, skilled nursing facility						

Additional Notes on Matching
The matching analysis was performed using SAS Enterprise Guide version 7.15 (SAS Institute Inc), using the PSMATCH procedure (optimal matching without replacement.

In the table above, we note that the post-match standardized mean difference (SMD) for some individual covariates exceeds one commonly used threshold to indicate negligible imbalance (0.10). We address this residual imbalance with the following points.

- **The overall imbalance between cohorts, as indicated by propensity score for participation in the outpatient BPCI-A BNESF episodes, is negligible.** This measure of balance across all covariates improves from 1.18 (pre-match) to 0.01 post-match, which is anticipated with the use of the optimal matching method. A plot of the area of common support for propensity scores is included in **Figure S5.**

- **For covariates where the SMD exceeds 0.10, literature suggests controlling for the covariate in the post-matching regression model, without issue up to an SMD of 0.25.** An SMD of 0.25 has been shown through simulation studies to maintain adequate balance with subsequent regression adjustment.

- **This and any additional residual imbalance were accounted for in subsequent models that estimate the primary and secondary outcomes.** Residual imbalance in hospital-level characteristics were adjusted by using hospital-level fixed effects in a sensitivity analysis, confirming the findings of the main model that only adjusted for patient and market characteristics. See **Supplement S7.**

References

1. Stuart EA. Matching methods for causal inference: a review and look forward. *Stat Sci*. 2010 February 1; 25(1): 1–21. doi:10.1214/09-STS313.
2. Rubin DB. Using Propensity Scores to Help Design Observational Studies: Application to the Tobacco Litigation. Health Services & Outcomes Research Methodology 2:169–188, 2001.
3. Haukoos JS, Lewis RJ. The Propensity Score. JAMA. 2015 Oct 20;314(15):1637-8.

© 2025 Kilaru AS et al. *JAMA Health Forum*.

B. Additional Matching Methods

Given the residual imbalance in the outpatient cohort after matching, we examined additional approaches to performing propensity score matching.

Given the relatively small sample size of treated individuals after the intervention in the outpatient cohort, we pre-specified that we would use 1:3 matching to optimize sample size. Compared to a stricter matching ratio (e.g. 1:1), the 2nd and 3rd match for each treated hospital may introduce bias; however, these additional matches also may decrease variance. Below, we demonstrate similarities in standardized mean difference regardless of matching ratio chosen; we include full results for the 1:1 match as a sensitivity analysis in **Supplement S11**.

Also below, we demonstrate the matching results for alternate matching strategies, including nearest neighbor matching (with replacement) and greedy matching (without replacement), again demonstrating little differences in the balance of participating and matched hospitals.

Table. Standardized mean differences for hospital characteristics after matching, compared across multiple matching ratios and methods

	**Standardized Mean Differences**					
	Before Matching	Optimal Matching 1:3	Optimal Matching 1:2	Optimal Matching 1:1	Nearest neighbor matching with replacement 1:3	Greedy nearest neighbor natching without replacement 1:3
Predicted Probability of BPCI-A Participation (Propensity Score)	1.18	**0.01**	0.005	0.005	0.06	0.01
**Ownership**, %						
Non-Profit	0.34	**0.21**	0.13	0.25	0.12	0.21
For-Profit	0.31	**0.24**	0.22	0.29	0.18	0.24
Government						
**Teaching status**, %						
Major	0.33	**0.08**	0.13	0.13	0.05	0.08
Minor	0.24	**0.23**	0.22	0.22	0.24	0.23
None						
**Annual Medicare days**, % of total inpatient days	0.51	**0.12**	0.11	0.16	0.02	0.12
**Total hospital bed count**, %						
Large	0.19	**0.00**	0.11	0.22	0.02	0.00
Medium	0.10	**0.17**	0.26	0.31	0.11	0.17
Small						
**Health system affiliation,** %						
Affiliated	0.03	**0.19**	0.32	0.38	0.31	0.19
None						
**Disproportionate share payment (2017),** mean, $	0.30	**0.11**	0.19	0.12	0.12	0.11
**BNESF volume (2017)**, mean, no.	0.17	**0.34**	0.58	0.19	0.36	0.34
**Annual hospital market share (2013 – 2018Q3)**, mean, %	0.34	**0.08**	0.09	0.06	0.07	0.08
**Discharges to highest volume SNF**, % of SNF discharges	0.16	**0.06**	0.06	0.42	0.03	0.06
**Population**, mean, no.	0.26	**0.21**	0.37	0.39	0.20	0.21
**Low-income status**, %	0.73	**0.03**	0.07	0.12	0.02	0.03
**SNF beds**, per 10000 Medicare beneficiaries	0.16	**0.07**	0.18	0.21	0.07	0.07
**Hospital Herfindahl-Hirschman Index**, score	0.65	**0.11**	0.13	0.11	0.10	0.11

© 2025 Kilaru AS et al. *JAMA Health Forum*.

C. Characteristics for inpatient participant and matched non-participant hospitals, pre/post matching

	Before Matching			After Matching		
	Participants (n = 41)	Non-Participants (n = 740)	Absolute Standard Mean Difference	Participants (n = 41)	Non-Participants (n = 123)	Absolute Standard Mean Difference
Predicted Probability of BPCI-A Participation (Propensity Score)	*see area of common support plot for distribution below*		0.81	*see area of common support plot for distribution below*		0.07
**Hospital-Level Characteristics**						
**Ownership**, %						
Non-Profit	82.9	69.6	0.31	82.9	78.0	0.12
For-Profit	12.2	19.2	0.19	12.2	17.9	0.16
Government	4.9	11.1		4.9	4.1	
**Teaching status**, %						
Major	26.8	20.7	0.14	26.8	26.8	0.00
Minor	58.5	59.1	0.01	58.5	54.5	0.08
None	14.6	20.1		14.6	18.7	
**Annual Medicare days**, % of total inpatient days	47.7	50.4	0.25	47.7	48.0	0.03
**Total hospital bed count**, %						
Large	39.0	41.9	0.06	39.0	43.1	0.08
Medium	58.5	50.9	0.15	58.5	52.0	0.13
Small	2.5	7.0		2.5	4.9	
**Health system affiliation,** %						
Affiliated	90.2	77.7	0.34	90.2	91.1	0.02
None	9.8	22.2		9.8	8.9	
**Urban status,** %						
Urban	100.0	99.0	--	100.0	100.0	--
Rural	0.0	1.0		0.0	0.0	

© 2025 Kilaru AS et al. *JAMA Health Forum*.

**Disproportionate share payment (2017),** mean, $	3,530,086	3,346,142	0.05	3,530,086	3,685,132	0.04
**BNESF volume (2017)**, mean, no.	29.7	27.3	0.11		30.2	0.02
**Annual hospital market share (2013 – 2018Q3)**, mean, %	17.2	19.7	0.14	17.2	15.8	0.08
**Discharges to highest volume SNF**, % of SNF discharges	20.6	21.5	0.09	20.6	20.6	0.01
**Hospital Market (Hospital Referral Region) Characteristics**						
**Population**, mean, no.	2,052,759	2,149,240	0.05	2,052,759	2,129,536	0.04
**Low-income status**, %	32.3	46.8	0.66	32.3	32.8	0.02
**SNF beds**, per 10000 Medicare beneficiaries	0.05	0.06	0.16	0.05	0.05	0.03
**Hospital Herfindahl-Hirschman Index**, score	1685	1965	0.19	1685	1544	0.09

BNESF, back and neck procedure except spinal fusion; SNF, skilled nursing facility

© 2025 Kilaru AS et al. *JAMA Health Forum*.

S5. Figure

A. Area of common support plot for propensity scores before and after matching – outpatient cohort

Before matching

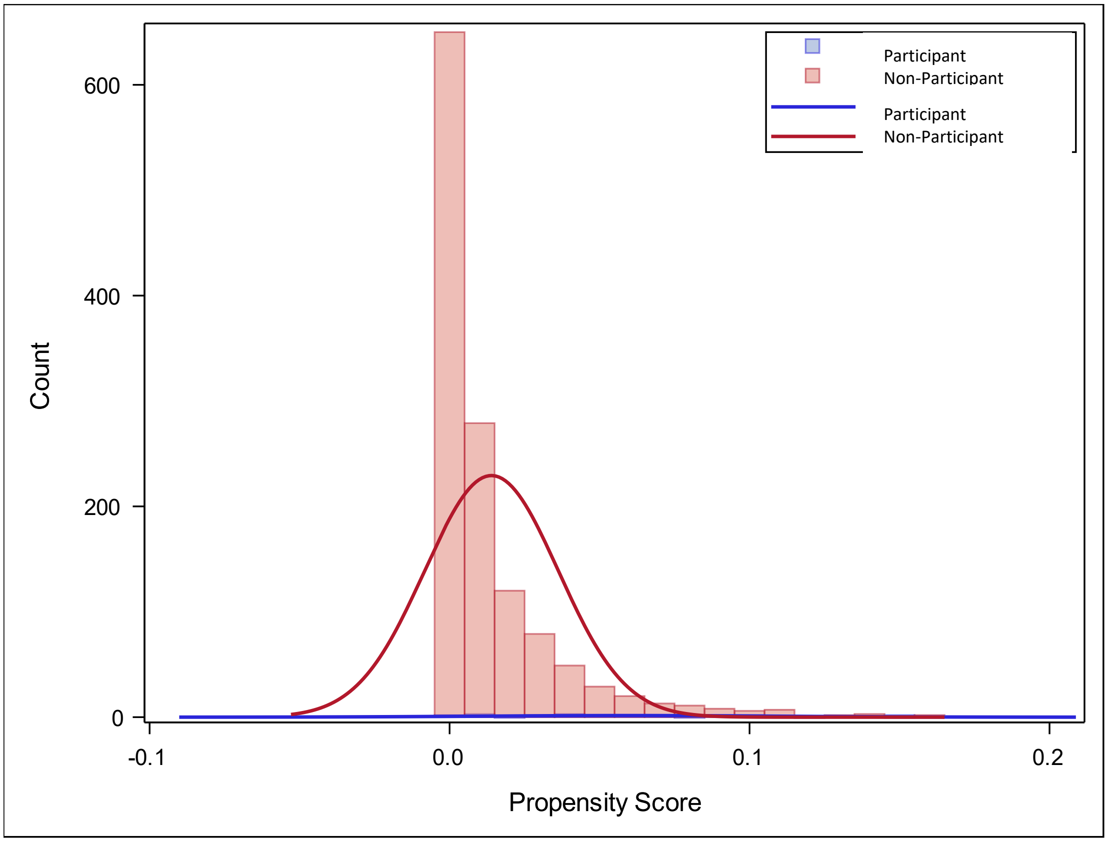

After matching

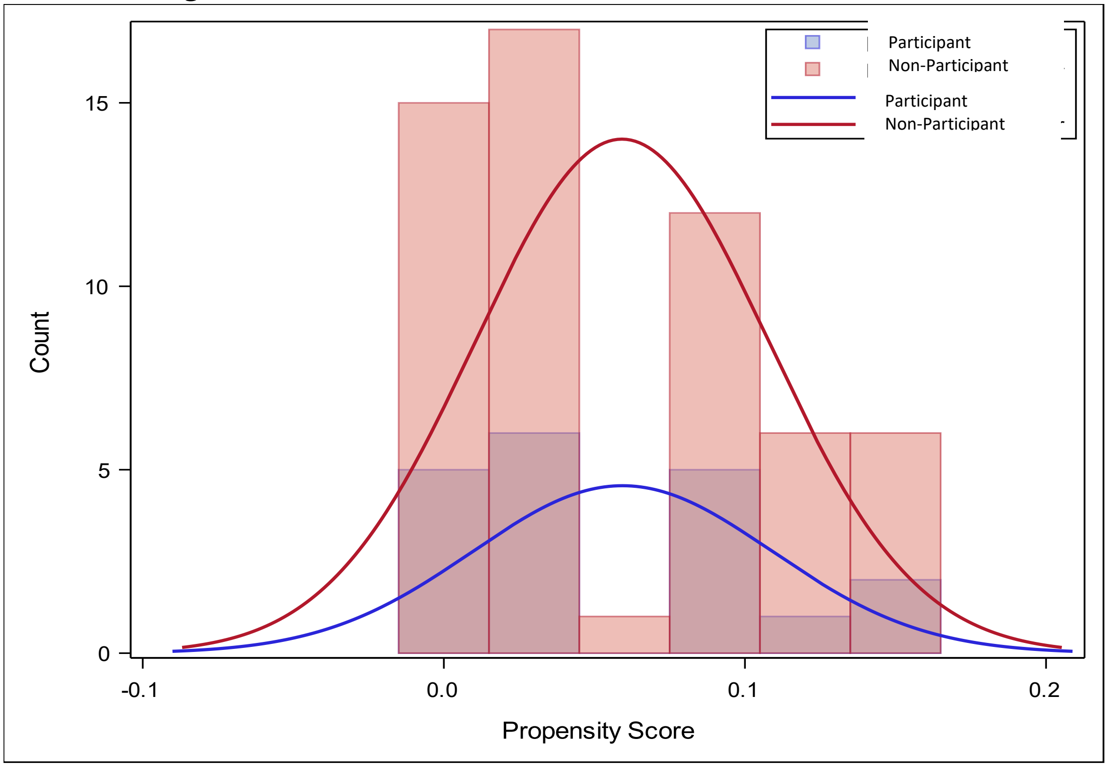

© 2025 Kilaru AS et al. *JAMA Health Forum*.

B. Area of common support plot for propensity scores before and after matching – inpatient cohort

Before matching

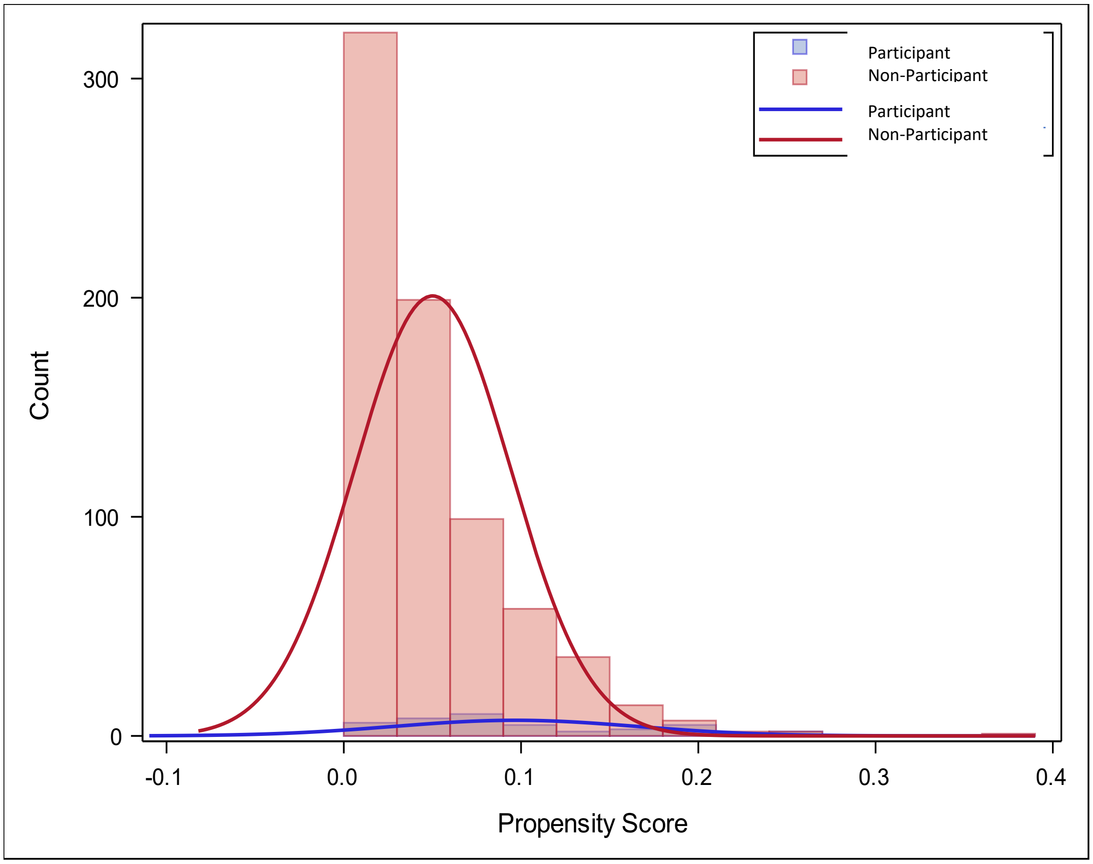

After matching

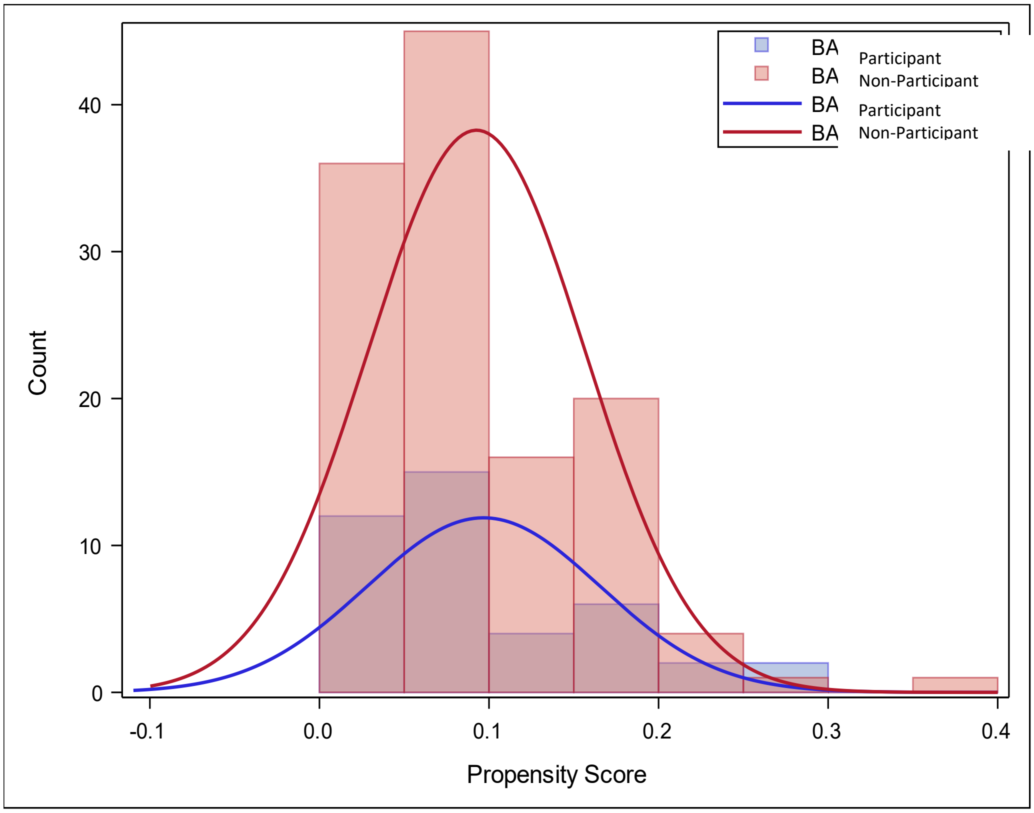

© 2025 Kilaru AS et al. *JAMA Health Forum*.

S6. Figure
Parallel trends for selected primary and secondary outcomes

The following plots demonstrate results of pre-period parallel trend testing. The plots were generated from models that test for divergent trends between participant and non-participant hospitals, by estimating each outcome as a function of time (quarter-years) and participant type. The pre-intervention period is shown; timepoints are presented in quarters (3 months). Point estimates represent the difference in the outcome between these groups as compared to the reference quarter, 2013 Q1. For any subsequent quarter, the difference is null if the 95% confidence interval (denoted by error bars) crosses zero.

As an additional analysis, we include models that estimate year-over-year, rather than quarter-year, differences between participant and non-participant hospitals, for the primary outcome, given that relatively few procedures are performed in each quarter-year interval leading to wide confidence intervals relative to the effect size. Results from this analysis were similar to our main analysis.

A. Outpatient Cohort

1. Total episode spending (primary outcome)

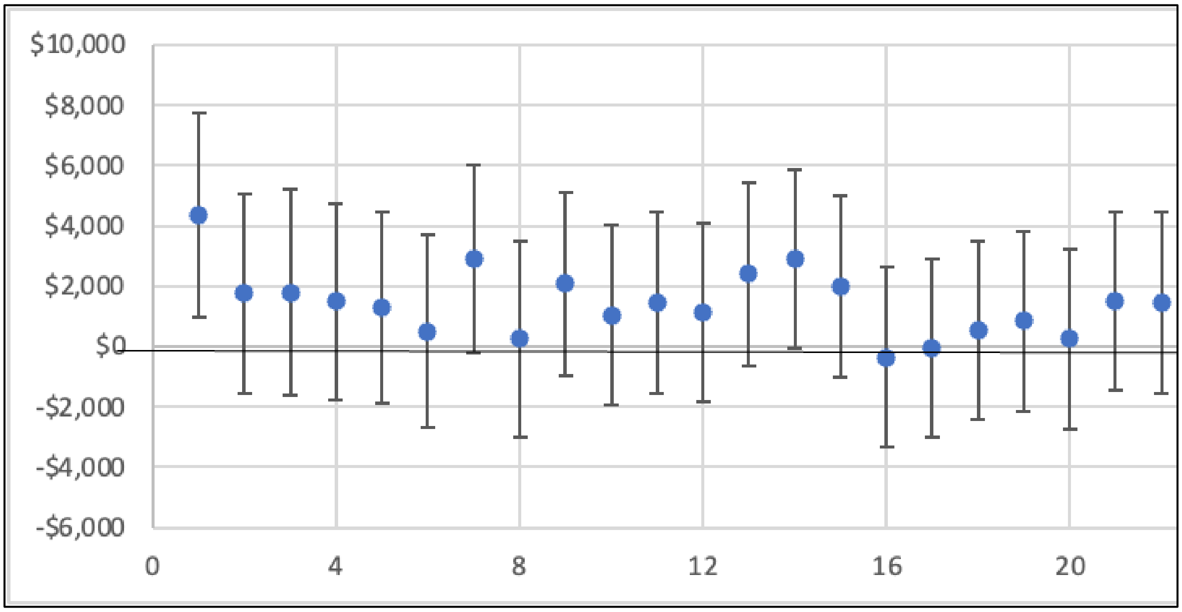

2. 90-day inpatient return admission

© 2025 Kilaru AS et al. *JAMA Health Forum*.

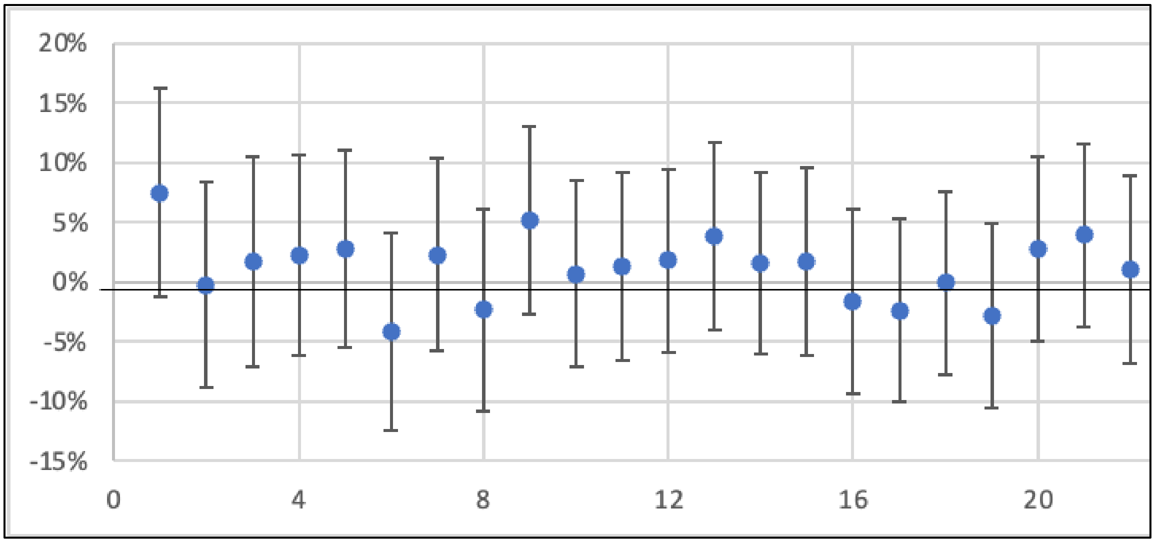

3. 90-day ED visit

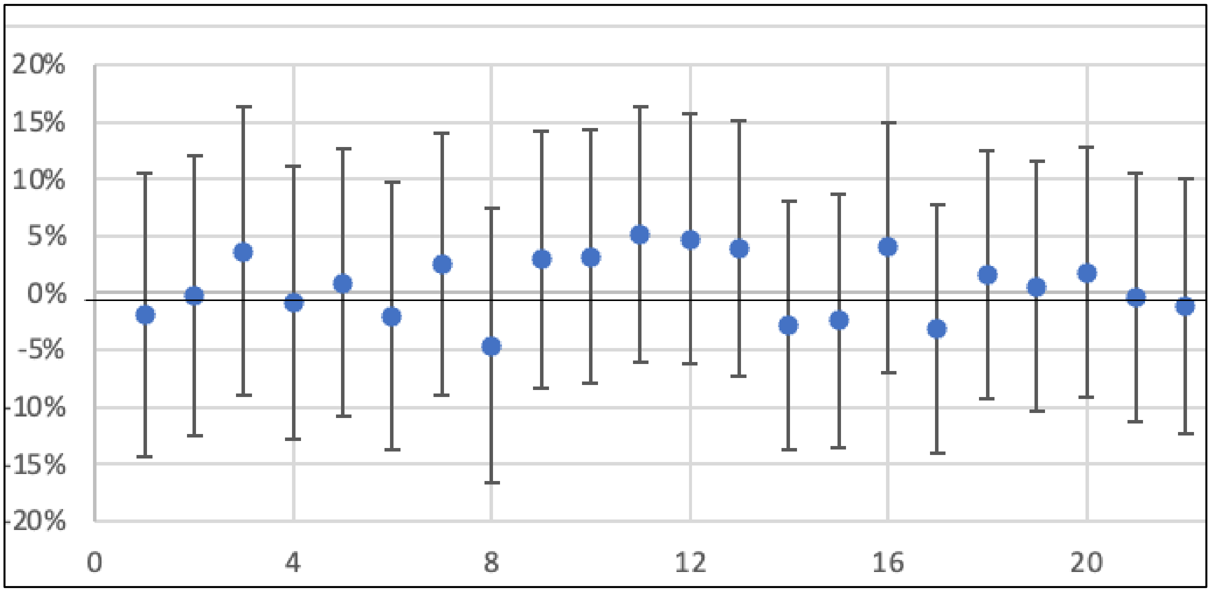

4. 90-day mortality

© 2025 Kilaru AS et al. *JAMA Health Forum*.

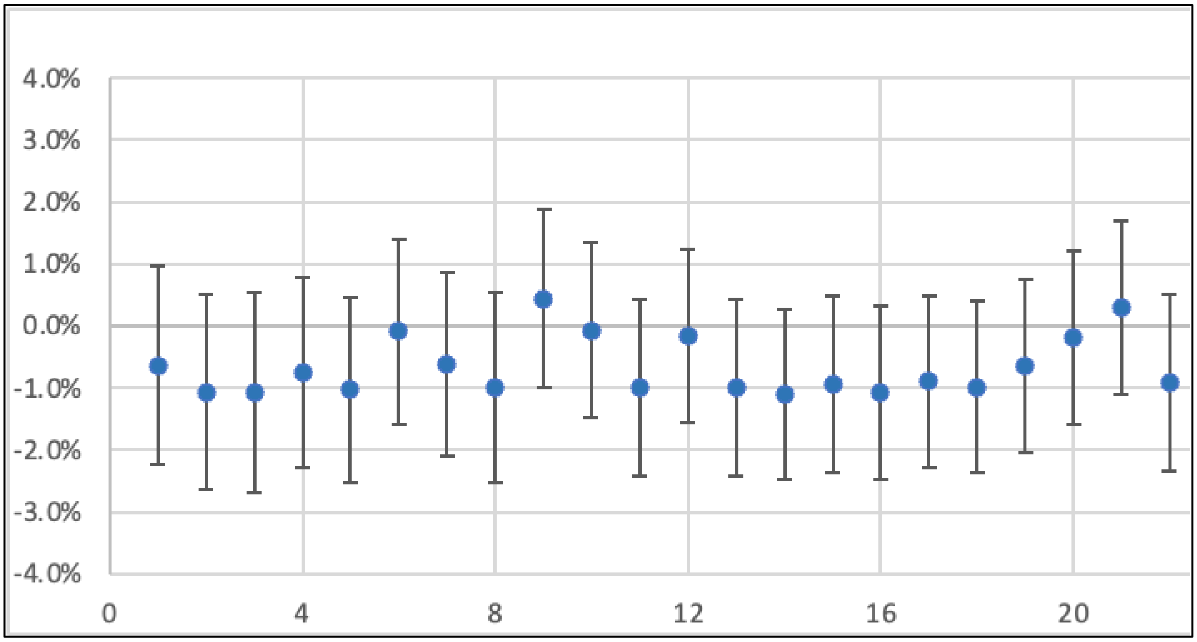

© 2025 Kilaru AS et al. *JAMA Health Forum*.

B. Inpatient Cohort

1. Total episode spending (primary outcome)

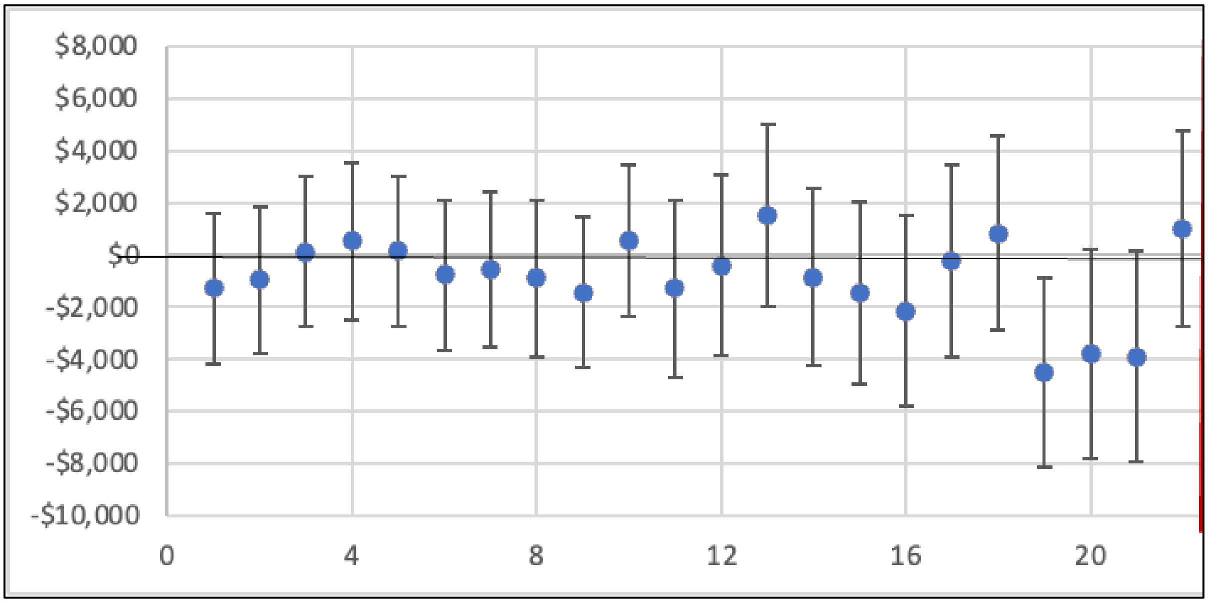

2. 90-day inpatient return admission

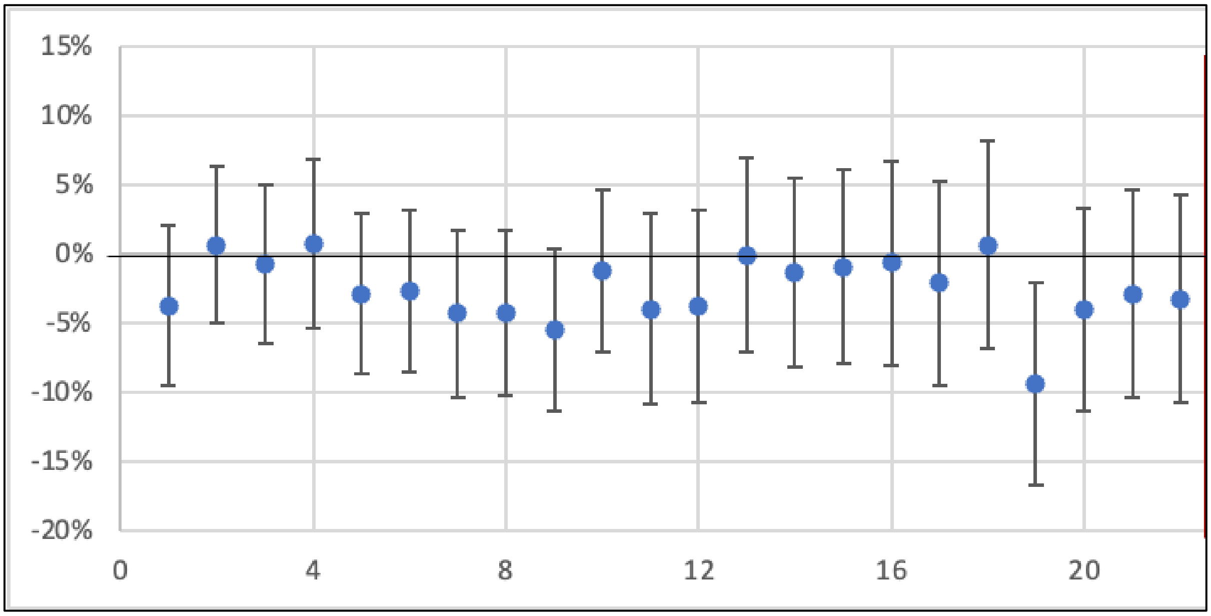

© 2025 Kilaru AS et al. *JAMA Health Forum*.

3. 90-day ED visit

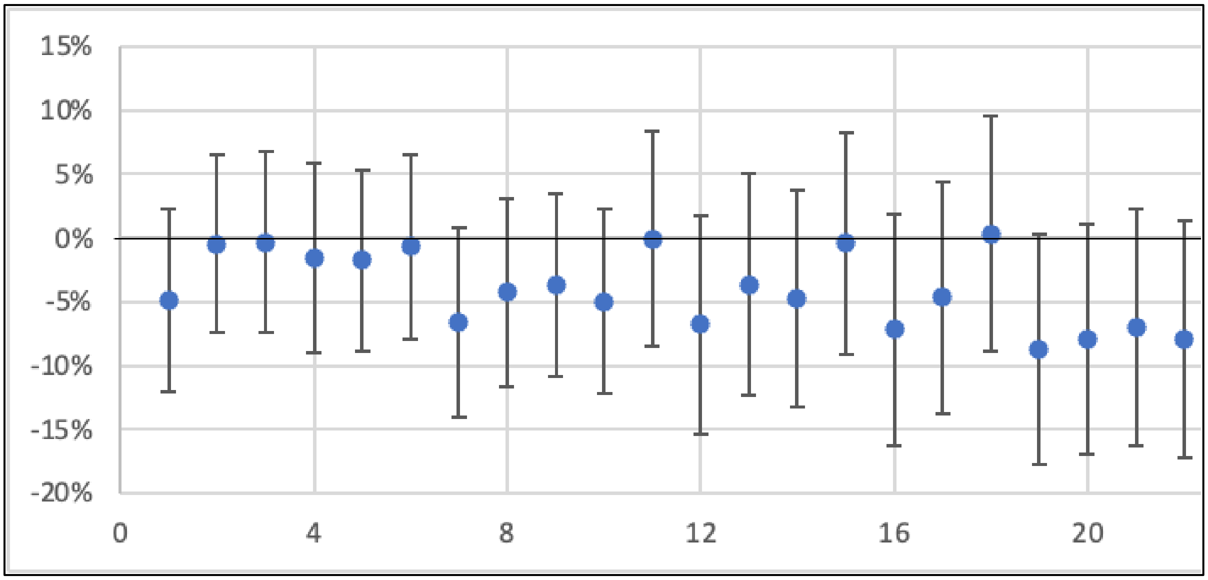

4. 90-day mortality

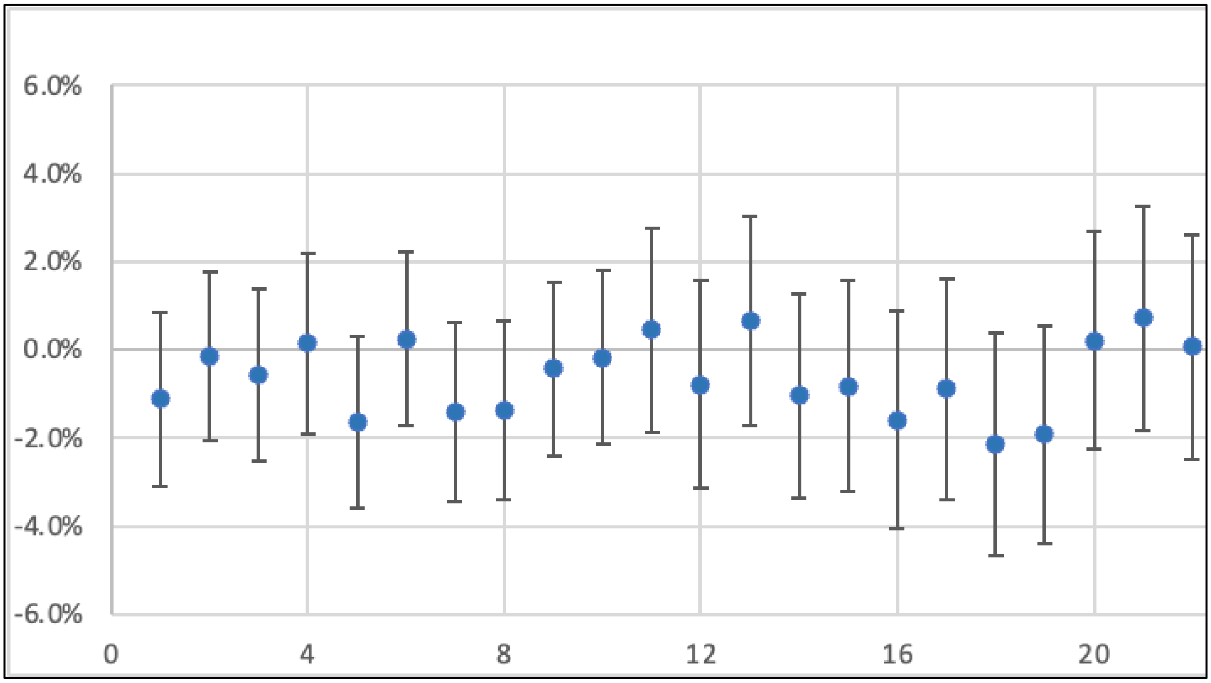

© 2025 Kilaru AS et al. *JAMA Health Forum*.

C. <u>Year-over-year trends</u>

Total episode spending – outpatient cohort

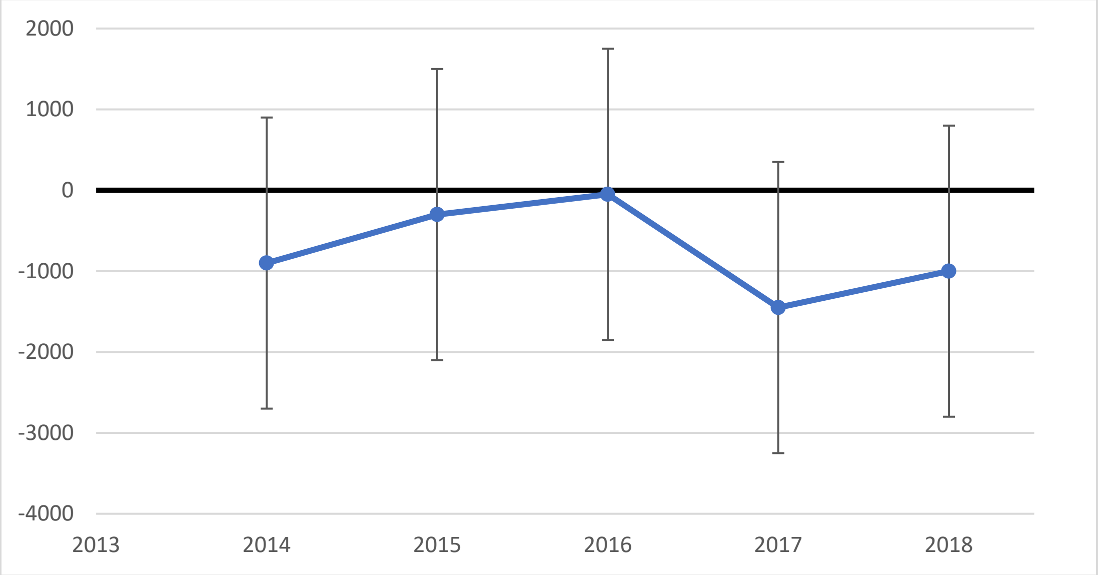

Total episode spending – inpatient cohort

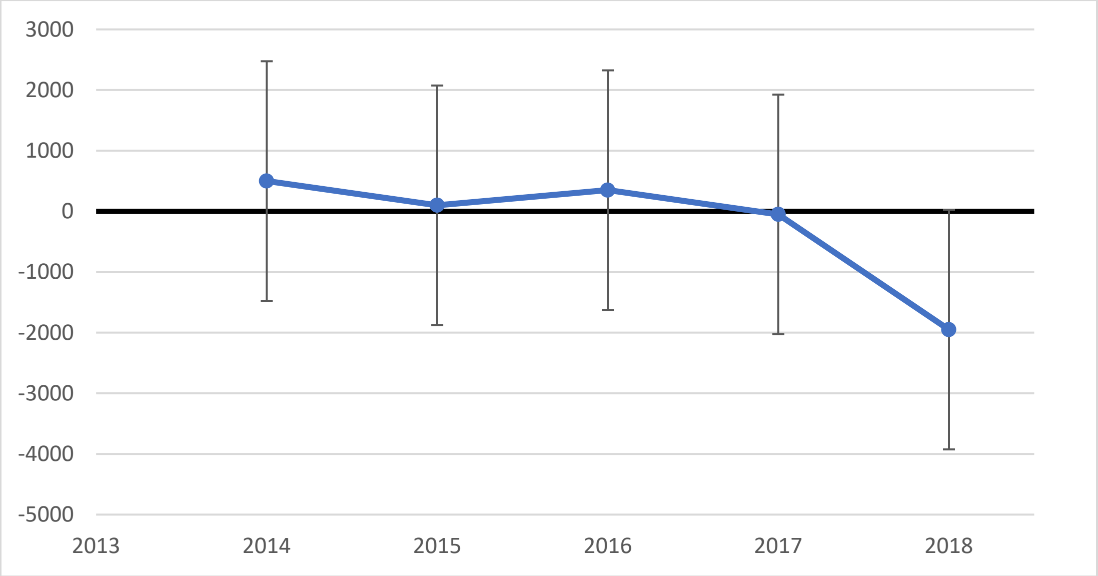

© 2025 Kilaru AS et al. *JAMA Health Forum*.

S7. Methods

Model specification

The following terms and equation summarize the differences-in-differences method used to estimate the adjusted changes in outcomes for beneficiaries receiving BNESF at hospitals with bundled payments as compared to non-participant hospitals, before and after implementation of bundled payments in BPCI Advanced. Separate analyses were performed for each outcome as well as for inpatient and outpatient cohorts.

Terms

Group	indicator of treatment group (participant or non-participant)
t	pre-post indicator of BPCI Advanced BNESF bundled payment participation
T	time fixed effect (quarter of admission date)
HRR	Hospital Referral Region for beneficiary fixed effect
Covar	patient-level covariates (including age, sex, race, ethnicity, disability status, dual status)
Comorbid	patient-level indicators for 1 of 29 Elixhauser co-morbidity conditions
Market	Hospital Referral Region characteristics, varying by quarter

- Size (total number of Medicare beneficiaries)
- Condition-specific (BNESF) total volume
- Medicare Advantage penetration
- ACO penetration
- Hospital Herfindahl-Hirschman index
- SNF Herfindahl-Hirschman index
- Total number of SNF beds

Inpatient episode analysis only:

Proc procedure fixed effect (MS-DRG 518, 519, 520; before 2014 Q1, DRG 490, 491)

Sensitivity analyses:

Hosp Hospital fixed effects

Model

$$Y_{i,h,q,m} = Group_i * t_h + Group_i + t_h + T_q + H_i + [\ Proc_i\ ] + Covar_i + Comorbid_i + Market_m + [Hosp_{\mathrm{h}}]$$

Clustered standard errors at hospital-level (independent correlation structure, identity link)

i, patient
h, hospital
q, quarter (time)
m, market (HRR)

© 2025 Kilaru AS et al. *JAMA Health Forum*.

S8. Table
Baseline market characteristics

The following tables summarizes market characteristics incorporated into the primary model. Please note the following considerations. First, these characteristics are time-varying. We describe characteristics for 2017, the year prior to BPCI Advanced. Second, there is overlap between market characteristics in the participant and non-participant study groups given that one region may include both types of hospitals. Third, additional variables were used in our approach for matching participant hospitals to non-participant hospitals; please see **Supplement S5**.

**Market characteristics (2017)**	**Outpatient Participant** (n = 15)	**Outpatient Non-Participant** (n = 34)	**Inpatient Participant** (n = 33)	**Inpatient Non-Participant** (n = 78)
**Accountable care organization penetration**, mean	31.8%	26.1%	29.7%	26.6%
**Medicare Advantage penetration**, mean	33.5%	36.3%	33.1%	30.7%
**Total skilled nursing facility beds**, mean	10721	14016	10859	9715
**Hospital Herfindahl-Hirschman index**, mean	1234	1152	1651	1906
**Skilled nursing facility Herfindahl-Hirschman index**, mean	301	271	351	398
**Number of Medicare beneficiaries**, mean	192477	234158	203172	186993
**Total volume of BNESF procedures**, mean	180	252	205	176

© 2025 Kilaru AS et al. *JAMA Health Forum*.

© 2025 Kilaru AS et al. *JAMA Health Forum*.

S9. Table
Episode spending, by category, for inpatient and outpatient episodes

Outpatient BNESF Episodes

Spending Categories	Participating Hospitals		Matched Non-Participating Hospitals		Adjusted Differences-in-Differences Estimate (95% CI)
	Before BPCI-A (n = 3155)	BPCI-A (n = 593)	Before BPCI-A (n = 8731)	BPCI-A (n = 1801)	
Total episode spending, dollars	$13100	$13086	$11677	$12946	-$1201 (-2184 to -219)
Professional services spending, dollars	$3969	$4088	$3605	$4000	-$245 (-466 to -24)
Total inpatient spending, dollars	$1873	$1472	$1236	$1524	-$675 (-1133 to -217)
Total outpatient spending, dollars	$6225	$6459	$6159	$6696	-$327 (-793 to 139)
Skilled nursing facility spending, dollars	$278	$342	$204	$135	$137 (-132 to 406)
Home health spending, dollars	$691	$638	$407	$514	-$105 (-278 to 69)

Inpatient BNESF Episodes

Spending Categories	Participating Hospitals		Matched Non-Participating Hospitals		Adjusted Differences-in-Differences Estimate (95% CI)
	Before BPCI-A (n = 3155)	BPCI-A (n = 593)	Before BPCI-A (n = 8731)	BPCI-A (n = 1801)	
Total episode spending, dollars	$26,581	$29,019	$25,075	$27,678	-$217 (-2334 to 1900)
Professional services spending, dollars	$5,439	$6,060	$5,226	$5,891	$41 (-411 to 494)
Total inpatient spending, dollars	$15,207	$16,866	$14,494	$15,785	$444 (-779 to 1666)
Total outpatient spending, dollars	$771	$1,071	$856	$1,013	$103 (-106 to 312)
Skilled nursing facility spending, dollars	$3,563	$3,166	$3,041	$3,211	-$711 (-1473 to 50)
Home health spending, dollars	$1,491	$1,750	$1,341	$1,631	-$59 (-345 to 227)

© 2025 Kilaru AS et al. *JAMA Health Forum*.

Trend plots for episode spending, by category (standardized payments, adjusted for inflation)

<u>Outpatient BNESF Episodes</u>

Intervention hospitals

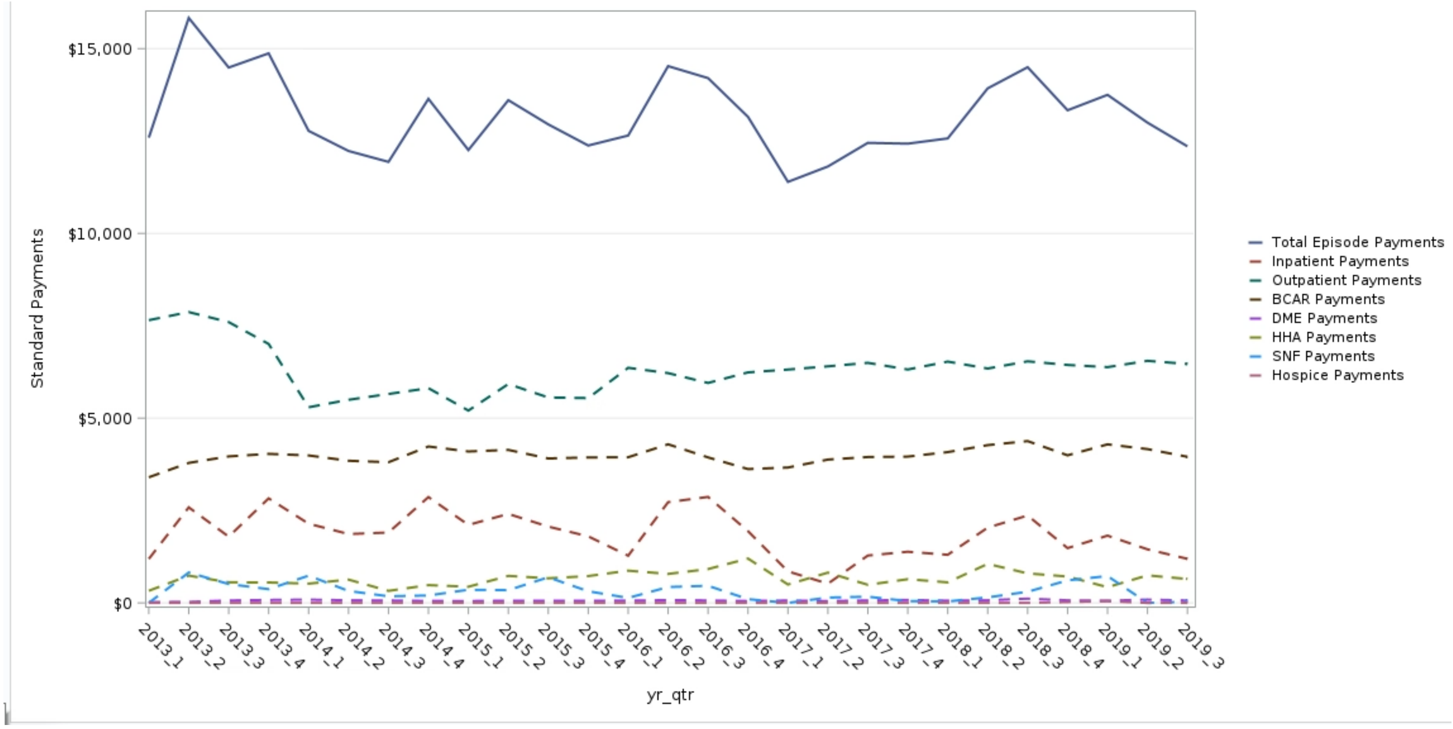

Comparison hospitals

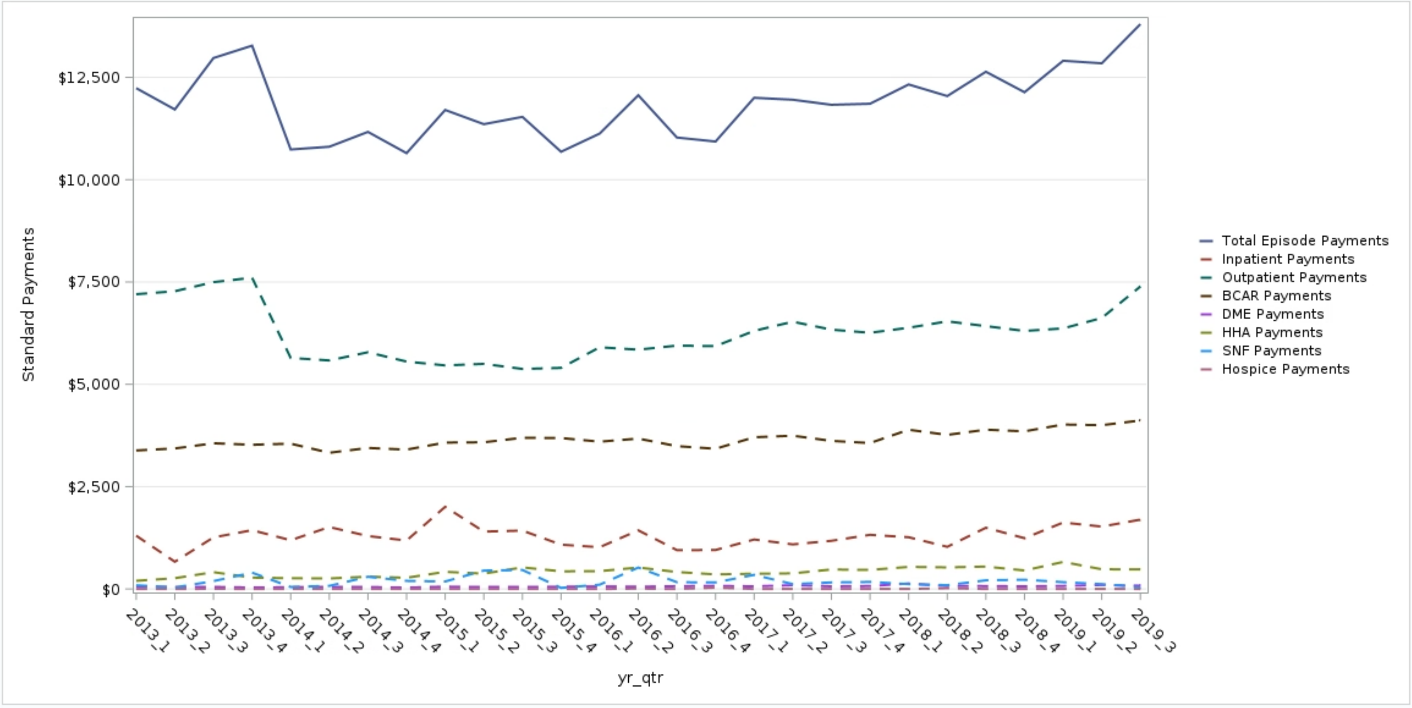

© 2025 Kilaru AS et al. *JAMA Health Forum*.

Inpatient BNESF Episodes

Intervention hospitals

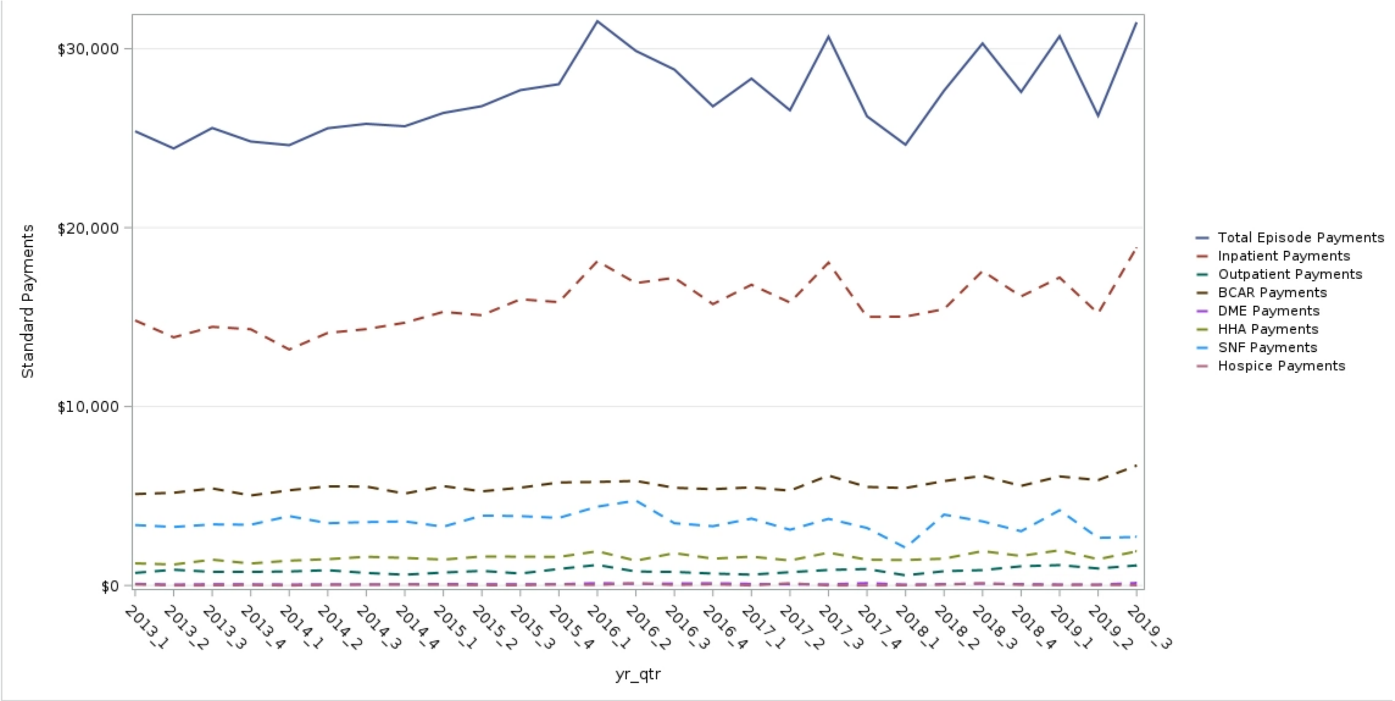

Comparison hospitals

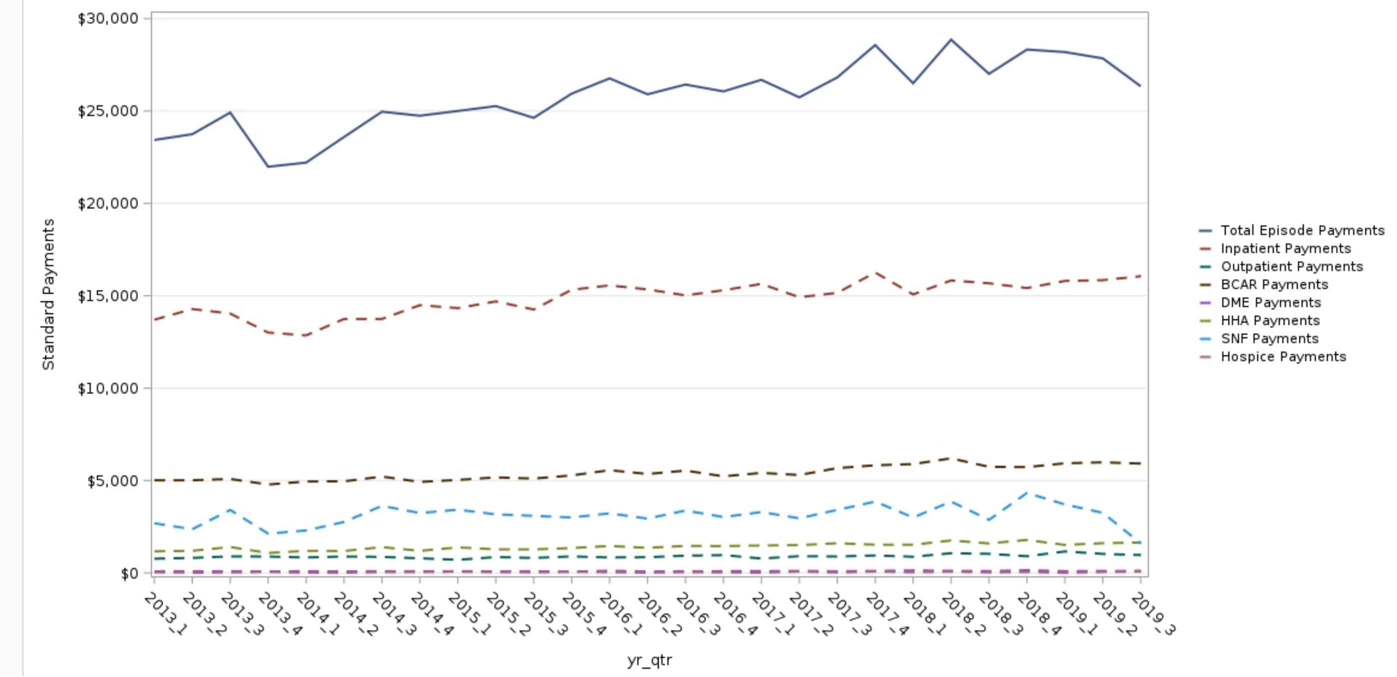

© 2025 Kilaru AS et al. *JAMA Health Forum*.

© 2025 Kilaru AS et al. *JAMA Health Forum*.

S10. Table
Return admissions occurring after index hospitalization, by DRG categories

Outpatient

	Participant				Non-Participant			
	Pre		Post		Pre		Post	
n	3155		593		8731		1801	
Total return admissions	256		26		602		142	
Other spine surgery, including spinal fusion	28	0.9%	4	0.7%	89	1.0%	12	0.7%
Other operative procedures	13	0.4%	2	0.3%	33	0.4%	13	0.7%
Repeat spine surgery without spinal fusion	7	0.2%	2	0.3%	21	0.2%	6	0.3%
Medical back problems or symptoms	27	0.9%	1	0.2%	63	0.7%	11	0.6%
Post-operative infection or sepsis	51	1.6%	9	1.5%	89	1.0%	29	1.6%

Inpatient cohort

	Participant				Non-Participant			
	Pre		Post		Pre		Post	
n	6142		622		15010		1666	
Total return admissions	860		107		2056		222	
Other spine surgery, including spinal fusion	75	1.2%	8	1.3%	198	1.3%	28	1.7%
Other operative procedures	42	0.7%	7	1.1%	116	0.8%	18	1.1%
Repeat spine surgery without spinal fusion	29	0.5%	3	0.5%	72	0.5%	7	0.4%
Medical back problems or symptoms	71	1.2%	8	1.3%	212	1.4%	18	1.1%
Post-operative infection or sepsis	184	3.0%	21	3.4%	406	2.7%	46	2.8%

DRG category groupings

- Other spine surgery, including spinal fusion
  - DRG 453, 454, 455, 456, 457, 458, 459, 460, 471, 472, 473, 029, 030
- Other operative procedures
  - DRG 463, 464, 465, 907, 908, 909, 515, 516, 517, 939, 940, 941
- Repeat spine surgery without spinal fusion
  - DRG 519, 520
- Medical back problems or symptoms
  - DRG 551, 552, 555, 556, 947, 948, 919, 920, 921
- Post-operative infection or sepsis
  - DRG 856, 857, 858, 862, 863, 870, 871, 872

© 2025 Kilaru AS et al. *JAMA Health Forum*.

© 2025 Kilaru AS et al. *JAMA Health Forum*.

S11. Table

Sensitivity analyses – differential changes in spending, quality, and utilization by hospital participation in BPCI-A outpatient BNESF episodes, with models that use

a) hospital fixed effects
b) 1:1 matching
c) multivariable regression modeling including all comparison hospitals, without matching
d) shorter pre-intervention period (starting in 2016 quarter 1)

A. Hospital fixed effects

Outpatient cohort

	**Adjusted Differences-in-Differences Estimate** (95% CI)
Total episode spending, dollars	-$1412 (-2393 to -432)
90-day inpatient readmission, %	-2.4 (-4.5 to -0.3)
90-day ED visit, %	0.7 (-3.2 to 4.6)
90-day mortality, %	-0.1 (-0.5 to 0.3)
7-day unplanned hospital visits, %	-2.7 (-6.7 to 1.3)
Discharge to post-acute care facility [b], %	-0.1 (-0.7 to 0.5)
Skilled nursing facility length-of-stay, days	0.2 (-0.2 to 0.6)
Discharge to home health, %	0.0 (-1.3 to 1.4)
14-day clinic visit, %	4.3 (-1.8 to 10.4)

Inpatient cohort

	**Adjusted Differences-in-Differences Estimate** (95% CI)
Total episode spending, dollars	-$298 (-2488 to 1892)
90-day inpatient readmission, %	3.2 (-0.6 to 7.0)
90-day ED visit, %	2.3 (-2.6 to 7.2)
90-day mortality, %	-0.03 (-1.4 to 1.4)
Discharge to post-acute care facility [b], %	-0.1 (-0.7 to 0.5)
Skilled nursing facility length-of-stay, days	-4.2 (-9.4 to 1.0)
Discharge to home health, %	3.2 (-1.0 to 7.3)
14-day clinic visit, %	0.4 (-4.4 to 4.8)

© 2025 Kilaru AS et al. *JAMA Health Forum*.

B. 1:1 Matching

Outpatient cohort

	**Adjusted Differences-in-Differences Estimate** (95% CI)
Total episode spending, dollars	-$1050 (-1907 to -193)
90-day inpatient readmission, %	-2.2 (-4.5 to -0.02)
90-day ED visit, %	2.7 (-1.2 to 6.7)
90-day mortality, %	-0.04 (-1.1 to 0.3)
7-day unplanned hospital visits, %	-1.6 (-5.2 to 2.1)
Discharge to post-acute care facility [b], %	-0.3 (-0.1 to 0.5)
Skilled nursing facility length-of-stay, days	0.2 (-0.2 to 0.7)
Discharge to home health, %	0.1 (-1.3 to 1.6)
14-day clinic visit, %	4.7 (-1.5 to 10.9)

Inpatient cohort

	**Adjusted Differences-in-Differences Estimate** (95% CI)
Total episode spending, dollars	-$187 (-2615 to 2240)
90-day inpatient readmission, %	3.4 (-0.7 to 7.4)
90-day ED visit, %	6.4 (1.2 to 11.6)
90-day mortality, %	0.3 (-1.4 to 1.9)
Discharge to post-acute care facility [b], %	-6.6 (-13.3 to -0.07)
Skilled nursing facility length-of-stay, days	-1.1 (-3.0 to 0.8)
Discharge to home health, %	0.1 (-1.3 to 1.6)
14-day clinic visit, %	-5.8 (-12.0 to 0.3)

© 2025 Kilaru AS et al. *JAMA Health Forum*.

C. Multivariable regression modeling with hospital fixed effects, without matching

Outpatient cohort

	**Adjusted Differences-in-Differences Estimate** (95% CI)
Total episode spending, dollars	-$612 (-1301 to 107)
90-day inpatient readmission, %	-1.1 (-2.7 to 0.4)
90-day ED visit, %	-0.9 (-4.1 to 2.4)
90-day mortality, %	-0.2 (-0.6 to 0.2)
7-day unplanned hospital visits, %	-3.1 (-6.6 to 0.3)
Discharge to post-acute care facility [b], %	-0.08 (-0.6 to 0.5)
Skilled nursing facility length-of-stay, days	0.2 (-0.3 to 0.8)
Discharge to home health, %	-0.03 (-1.4 to 1.3)
14-day clinic visit, %	2.8 (-2.8 to 8.3)

Inpatient cohort

	**Adjusted Differences-in-Differences Estimate** (95% CI)
Total episode spending, dollars	-$333 (-2379 to 1741)
90-day inpatient readmission, %	2.1 (-1.4 to 5.6)
90-day ED visit, %	1.8 (-2.5 to 6.0)
90-day mortality, %	0.2 (-1.0 to 1.4)
Discharge to post-acute care facility [b], %	-3.4 (-8.0 to 1.2)
Skilled nursing facility length-of-stay, days	-1.0 (-2.1 to 0.2)
Discharge to home health, %	3.6 (-0.2 to 7.3)
14-day clinic visit, %	-0.4 (-4.1 to 4.9)

© 2025 Kilaru AS et al. *JAMA Health Forum*.

D. Shorter pre-intervention pre-period (starting in 2016 Q1)

Outpatient cohort

	**Adjusted Differences-in-Differences Estimate** (95% CI)
Total episode spending, dollars	-$1149 (-2257 to -41)
90-day inpatient readmission, %	-2.7 (-5.7 to 0.1)
90-day ED visit, %	0.9 (-3.5 to 5.3)
90-day mortality, %	-0.2 (-0.8 to 0.4)
7-day unplanned hospital visits, %	-2.5 (-6.0 to 1.0)
Discharge to post-acute care facility [b], %	0.06 (-0.06 to 0.7)
Skilled nursing facility length-of-stay, days	0.3 (-0.1 to 0.7)
Discharge to home health, %	0.1 (-1.5 to 1.7)
14-day clinic visit, %	1.6 (-4.5 to 7.6)

Inpatient cohort

	**Adjusted Differences-in-Differences Estimate** (95% CI)
Total episode spending, dollars	$209 (-1992 to 2411)
90-day inpatient readmission, %	3.9 (-0.6 to 8.3)
90-day ED visit, %	5.1 (-0.2 to 10.4)
90-day mortality, %	0.1 (-1.3 to 1.6)
Discharge to post-acute care facility [b], %	-2.6 (-7.6 to 2.3)
Skilled nursing facility length-of-stay, days	-0.4 (-1.8 to 1.0)
Discharge to home health, %	2.0 (-2.6 to 6.6)
14-day clinic visit, %	0.1 (-6.2 to 6.4)

© 2025 Kilaru AS et al. *JAMA Health Forum*.

© 2025 Kilaru AS et al. *JAMA Health Forum*.

S12. Table
Analysis for patient selection effects: difference-in-difference analysis of key patient characteristics

The following tables present the results of an analysis to examine for potential patient selection following participation in the BPCI-A model. We used a differences-in-differences approach to estimate changes in each of the following observed patient characteristics, comparing participant hospitals to matched non-participant hospitals, following the start of BPCI-A in 2018 Q4. We conducted separate analyses for outpatient and inpatient episodes. This analysis does not account for any patient selection that may have occurred due to unobserved factors.

These results indicate that there was little evidence of patient selection according to these patient-level characteristics, with regard to the characteristics of patients that received outpatient surgeries or inpatient surgeries. There was a significant increase in the percentage of dual-eligible patients that received outpatient BNESF procedures. Given that dual eligibility is often associated with higher medical and social complexity, this increase is unlikely to bias the main findings that outpatient episodes had reduced spending.

Outpatient Episodes

Patient characteristics	Adjusted Differences-in-Differences Estimate (95% CI)	*P* value
**Age**, years	-0.02 (-0.89 to 0.85)	0.97
**Female**, %	-1.0 (-6.1 to 4.0)	0.69
**Black race**, %	0.8 (-1.1 to 2.8)	0.40
**White race**, %	-1.4 (-4.7 to 2.0)	0.42
**Hispanic ethnicity**, %	-0.6 (-2.4 to 1.2)	0.52
**Dual eligible, %**	3.5 (0.7 to 6.4)	0.01
**Disabled**, %	0.7 (-2.4 to 3.8)	0.66
**Prior hospitalization in past 1 year,** %	-2.7 (-6.1 to 0.8)	0.13
**Prior skilled nursing facility in past 1 year,** %	-0.6 (-2.0 to 0.7)	0.35
**Elixhauser co-morbidity score**	-0.03 (-0.9 to 0.8)	0.95
**Total number of Elixhauser comorbidities**, n	0.009 (-0.03 to 0.05)	0.52

Inpatient Episodes

Patient characteristics	Adjusted Differences-in-Differences Estimate (95% CI)	*P* value
**Age**, years	-0.02 (-0.9 to 0.9)	0.96
**Female**, %	2.2 (-2.6 to 7.0)	0.37
**Black race**, %	-0.4 (-2.4 to 1.6)	0.71
**White race**, %	1.1 (-2.0 to 4.2)	0.48
**Hispanic ethnicity**, %	0.7 (-1.0 to 2.3)	0.42
**Dual eligible, %**	1.5 (-1.6 to 4.5)	0.35
**Disabled**, %	0.1 (-2.9 to 3.2)	0.94
**Prior hospitalization in past 1 year,** %	3.7 (-0.3 to 7.6)	0.06
**Prior skilled nursing facility in past 1 year,** %	-0.5 (-2.6 to 1.6)	0.64
**Elixhauser co-morbidity score**	0.7 (-0.4 to 1.7)	0.22
**Total number of Elixhauser comorbidities**, n	0.3 (-0.01 to 0.5)	0.06
**Index Hospitalization length-of-stay**, days	0.02 (-0.25 to 0.30)	0.86

© 2025 Kilaru AS et al. *JAMA Health Forum*.

S13. Table

Analysis for patient selection effects – analysis of shifts in volume between inpatient and outpatient surgeries

The previous analysis for selection did not examine whether participants were offered the procedure at the same rate, and whether there were shifts in volume between inpatient and outpatient procedures. In this set of plots, we an additional examination of these potential shifts.

First, we plotted the unadjusted volume of inpatient and outpatient procedures for 6 different groupings of participants (these are non-mutually exclusive):

1) All hospital participants in either inpatient OR outpatient OR both (all)
2) Hospital participants in either outpatient OR both (all outpatient)
3) Hospital participants in either inpatient OR both (all inpatient)
4) Hospital participants in both inpatient and outpatient ONLY
5) Hospital participants in outpatient ONLY
6) Hospital participants in inpatient ONLY

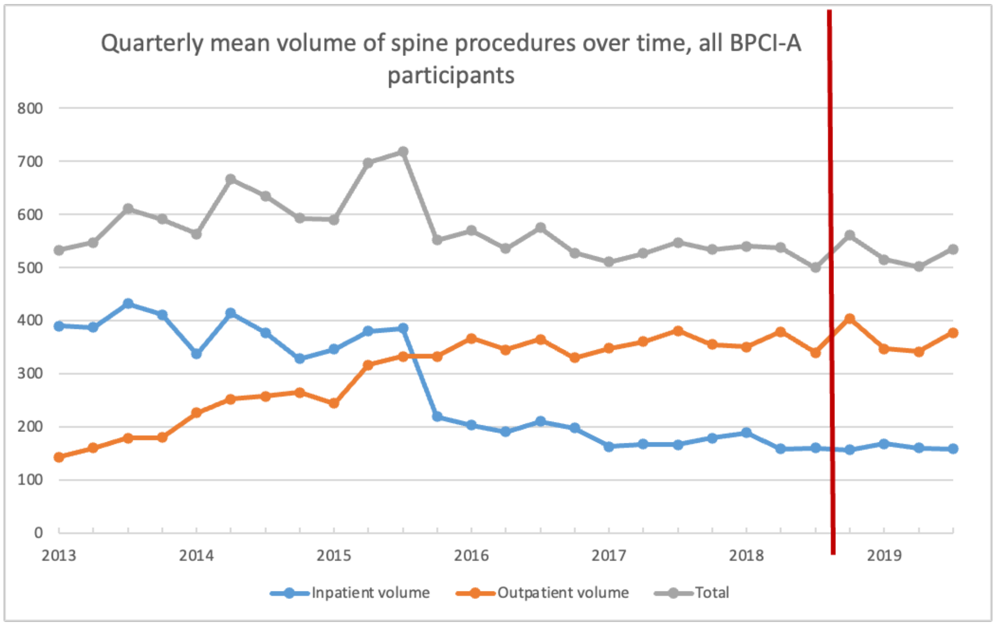

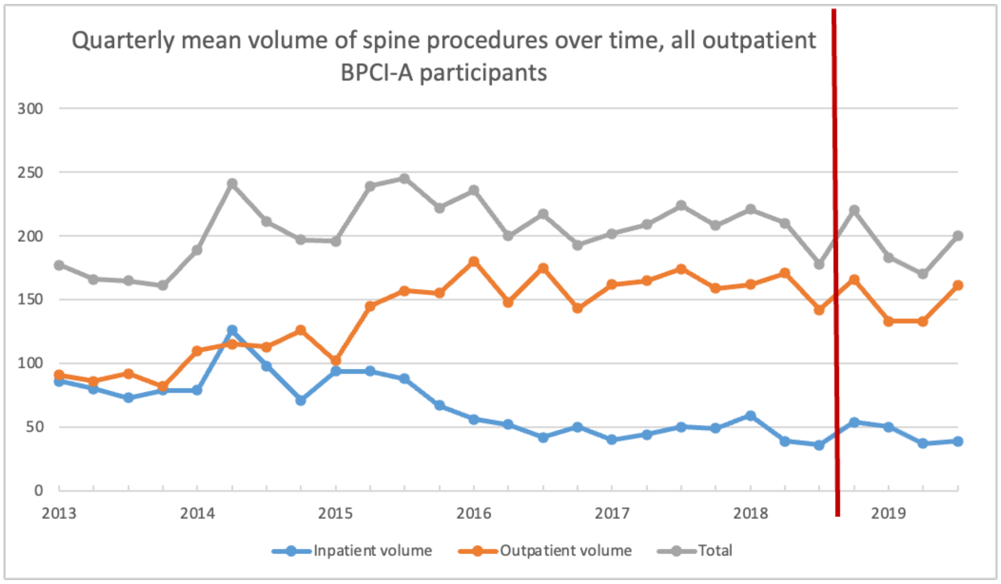

© 2025 Kilaru AS et al. *JAMA Health Forum*.

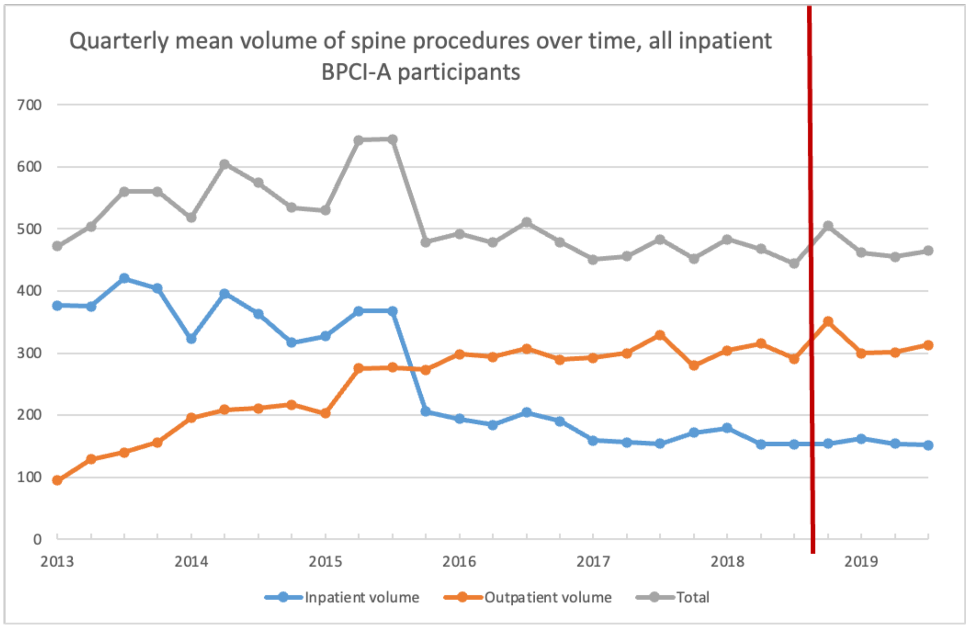

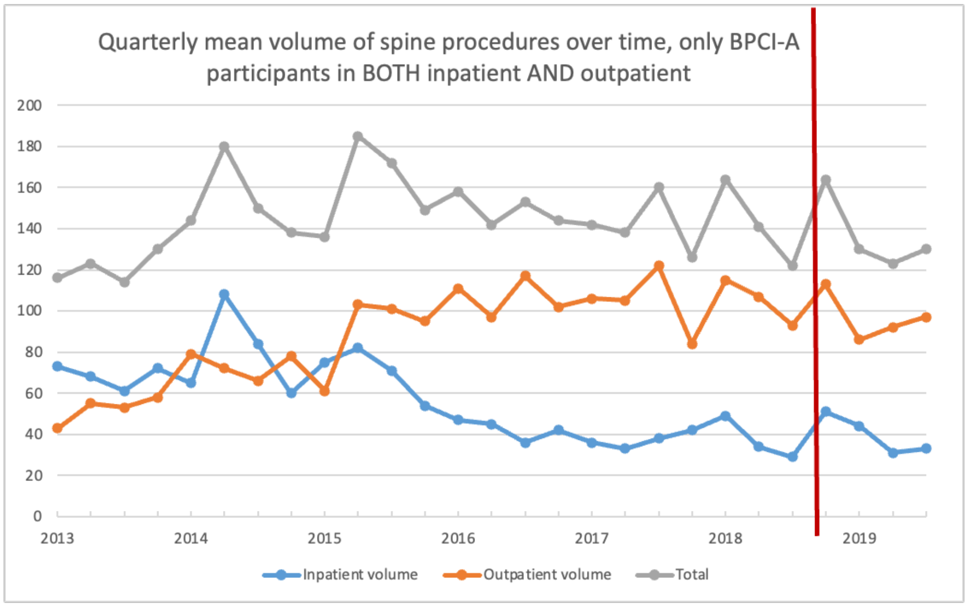

© 2025 Kilaru AS et al. *JAMA Health Forum*.

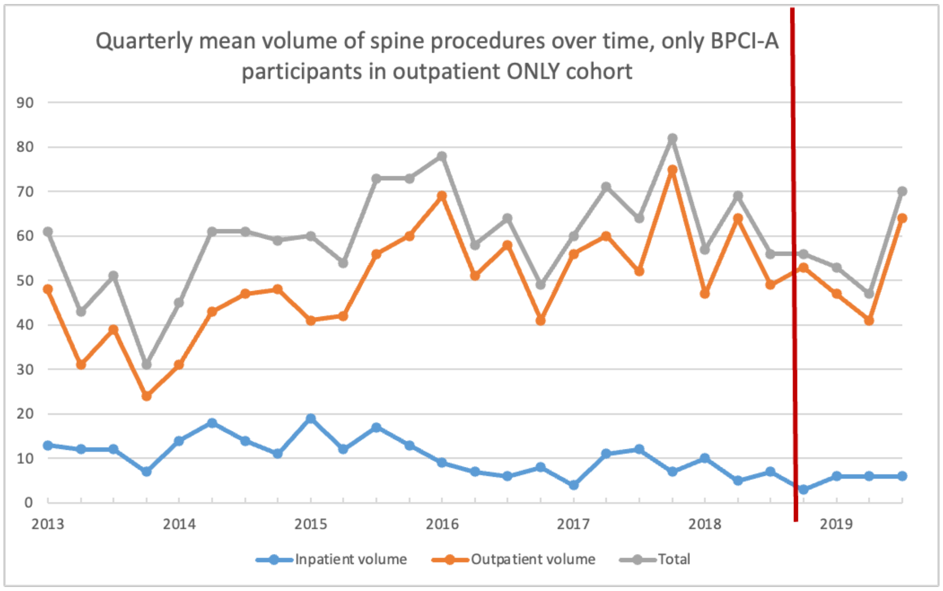

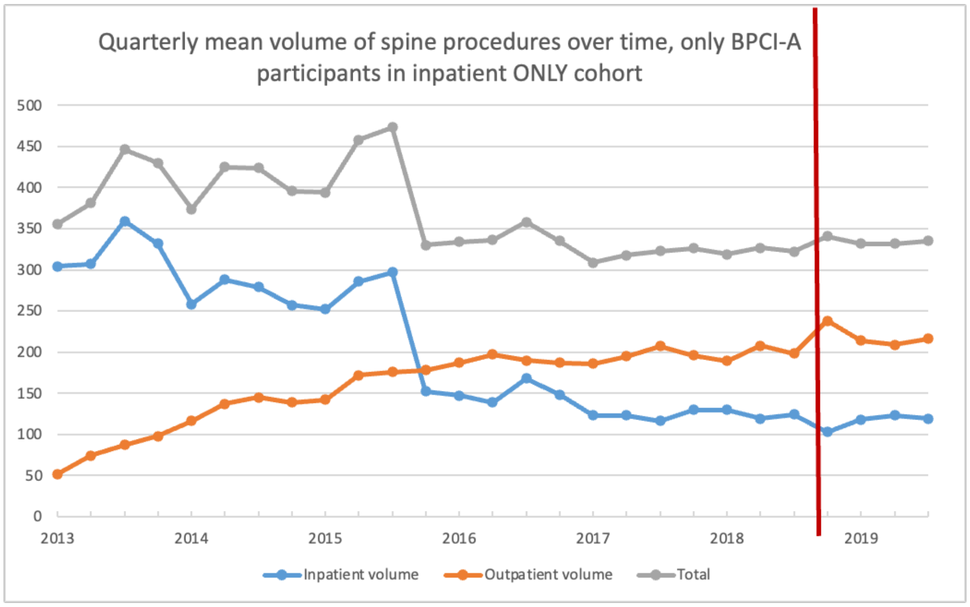

Reviewing these plots, there was no apparent evidence of changes to either inpatient volume or outpatient volume when examining Group 1-3 and Group 6, comparing before and after the intervention. For Group 4, there is a possible suggestion of a trend that volumes for both inpatient surgeries and outpatient surgeries declined after the intervention. For Group 5, we found suggestion of a trend that volumes for outpatient episodes increased after the intervention.

We ran additional regression models for Groups 4-6 to determine whether any trends were statistically significant. In these models, the outcomes were the volume of 1) outpatient, 2) inpatient, or 3) all surgeries, with covariate being time. In these plots, error bars denote 95% confidence intervals relative to the reference period (2013 Q1), although we are specifically examining for overlap in confidence intervals before and after the intervention (2018 Q1). While there are sustained shifts in volumes over the entire study period due to previously described trends in the increase of outpatient and decrease of inpatient procedures, there do not appear to be significant changes in either inpatient or outpatient volume immediately following the intervention in 2018 Q1, regardless of Group.

Group 4 – Both Inpatient and Outpatient Episode Participants

Outpatient surgery volume, by quarter

© 2025 Kilaru AS et al. *JAMA Health Forum*.

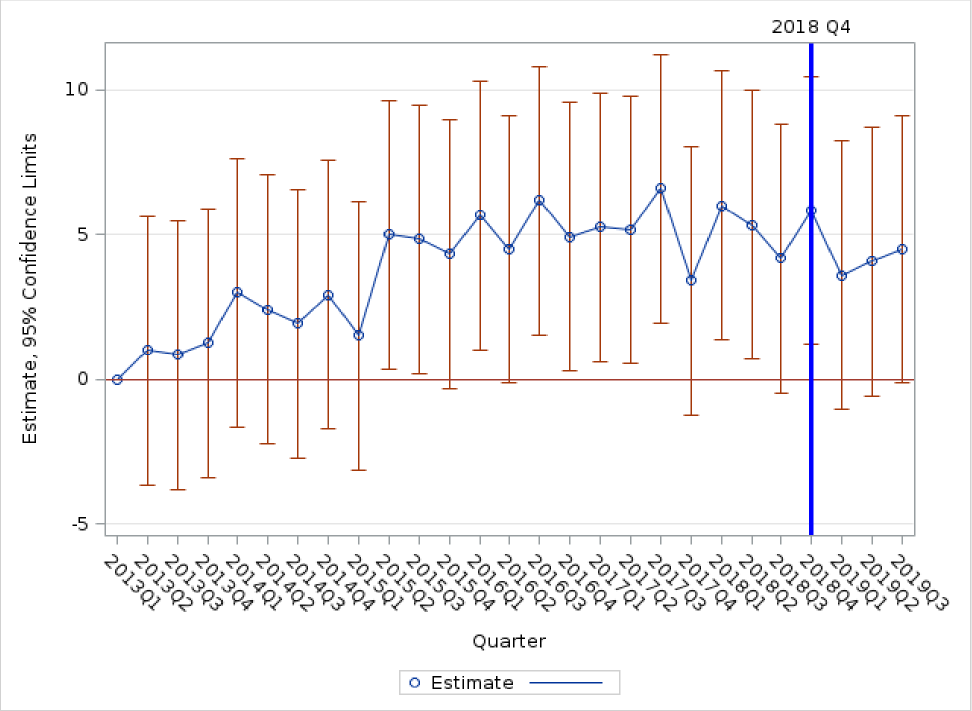

Inpatient surgery volume, by quarter

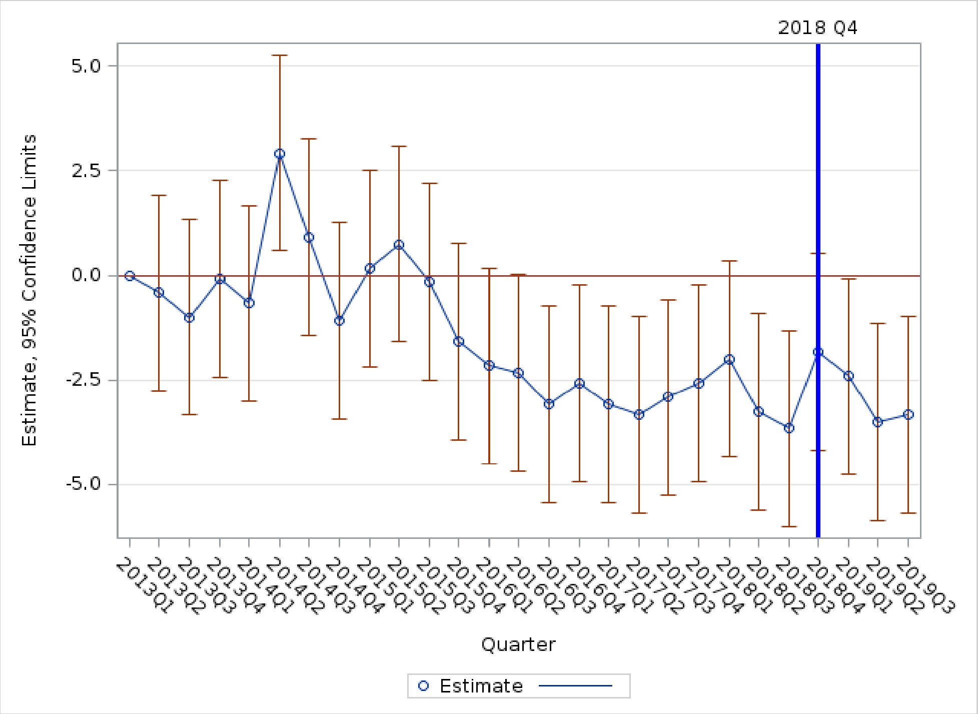

All surgery volume, by quarter

© 2025 Kilaru AS et al. *JAMA Health Forum*.

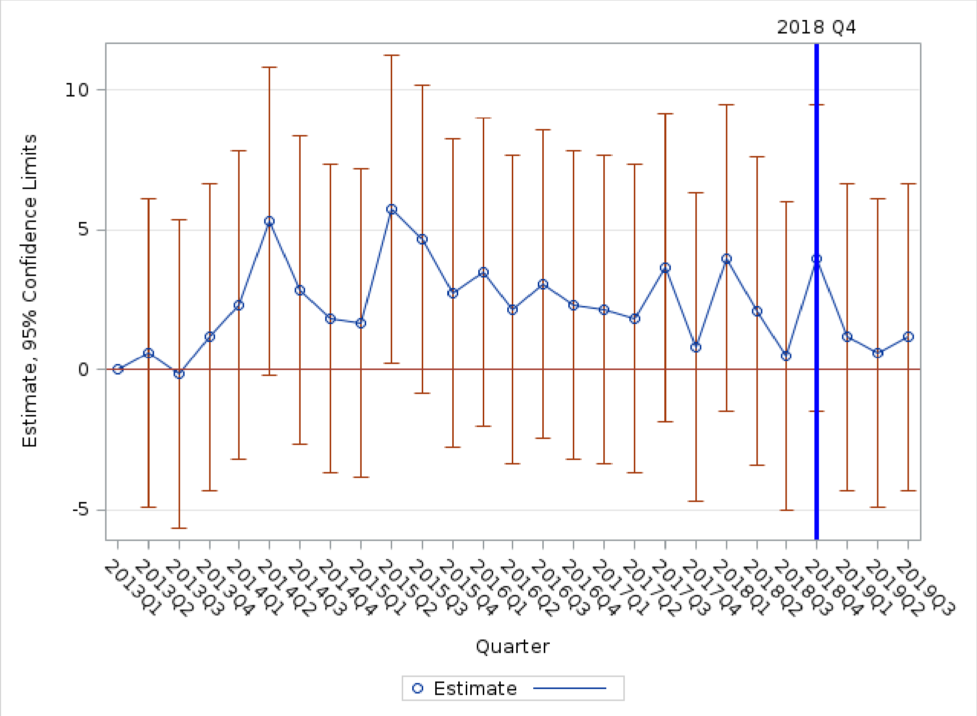

Group 5 – Outpatient Episode Participants Only

Outpatient surgery volume, by quarter

© 2025 Kilaru AS et al. *JAMA Health Forum*.

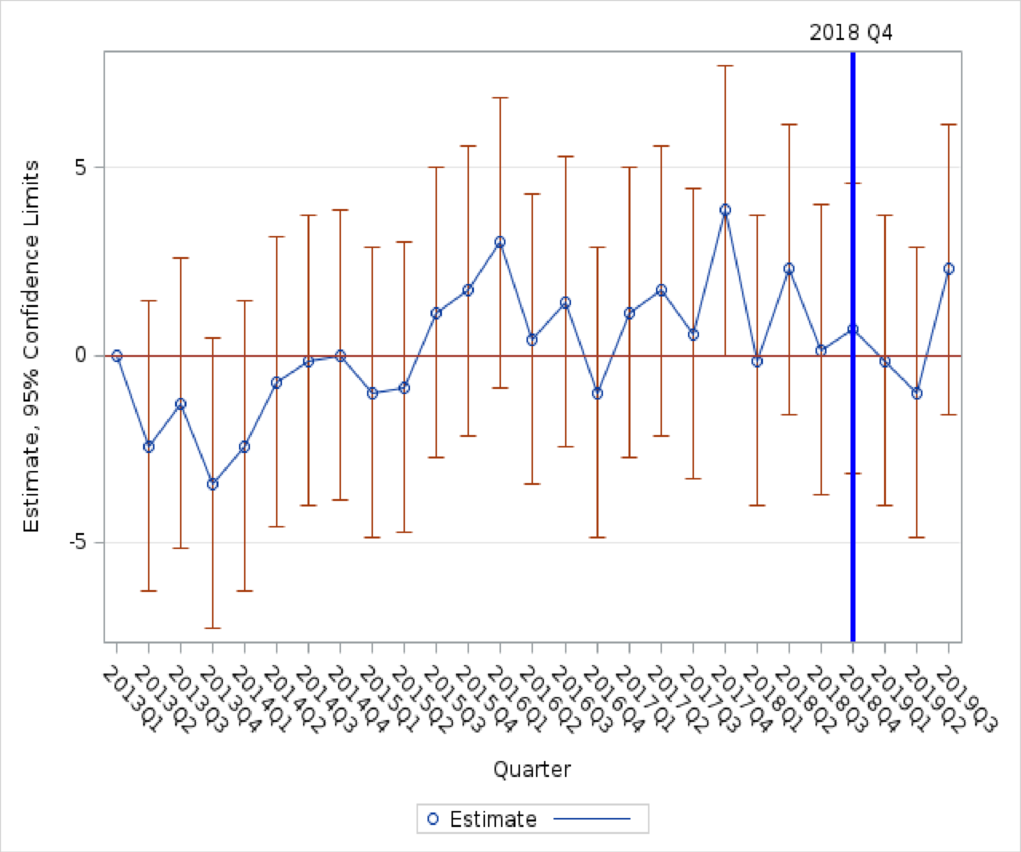

Inpatient surgery volume, by quarter

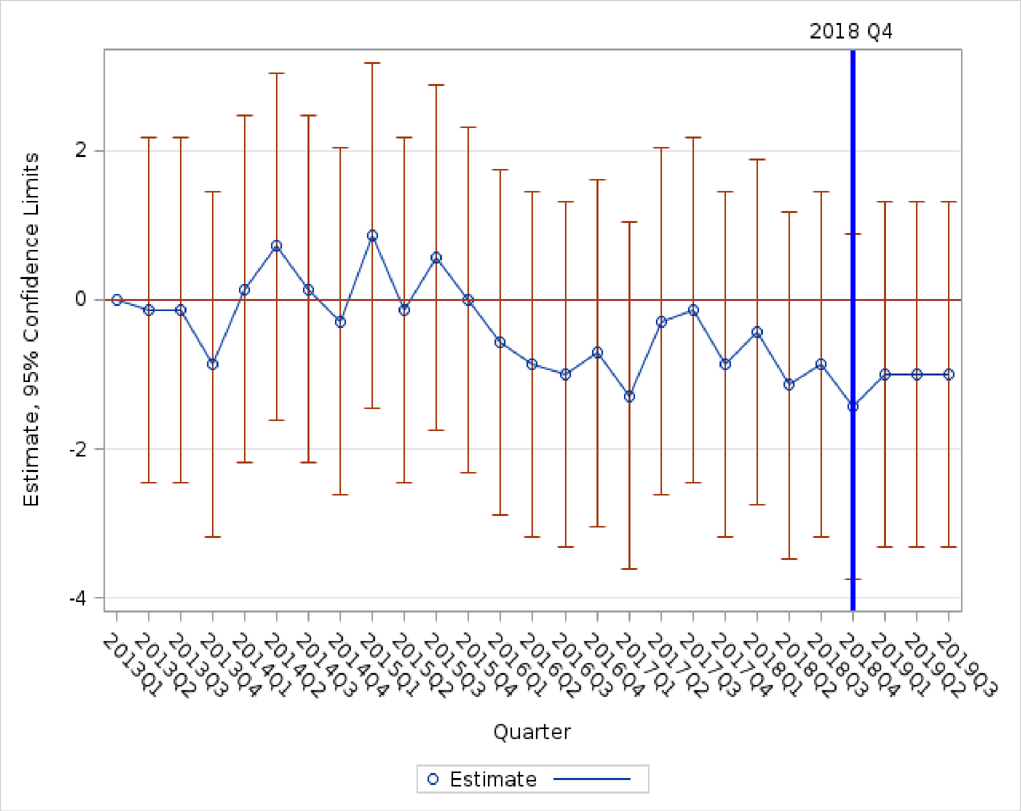

All surgery volume, by quarter

© 2025 Kilaru AS et al. *JAMA Health Forum*.

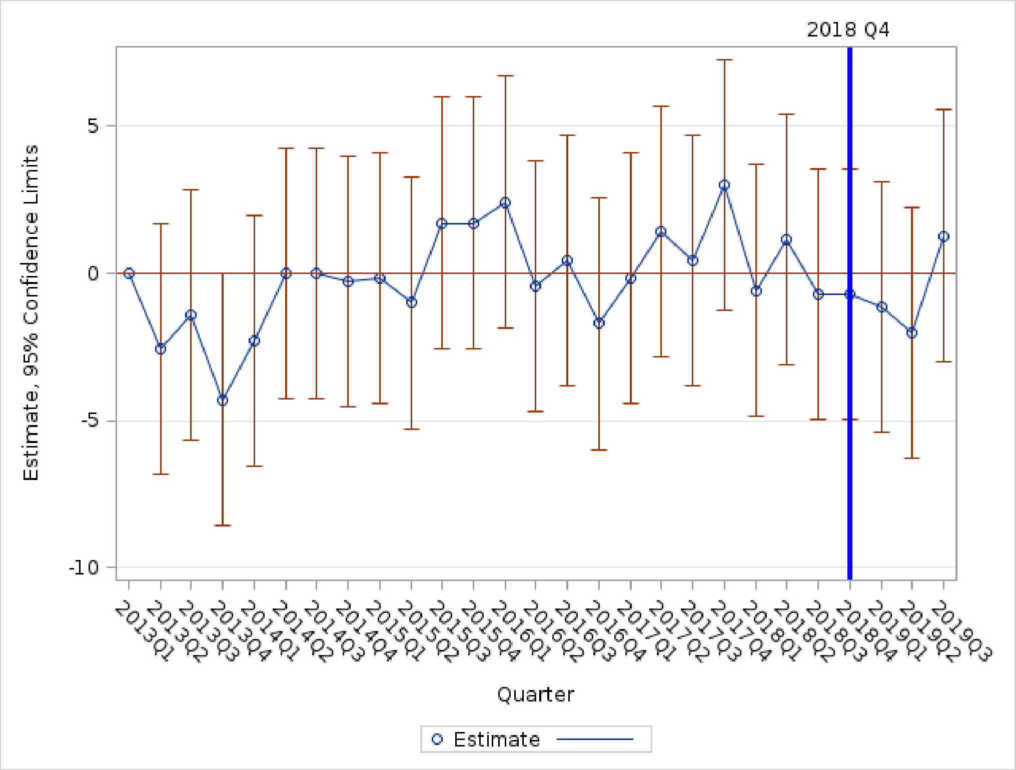

Group 6 – Inpatient Episode Participants Only

Outpatient surgery volume, by quarter

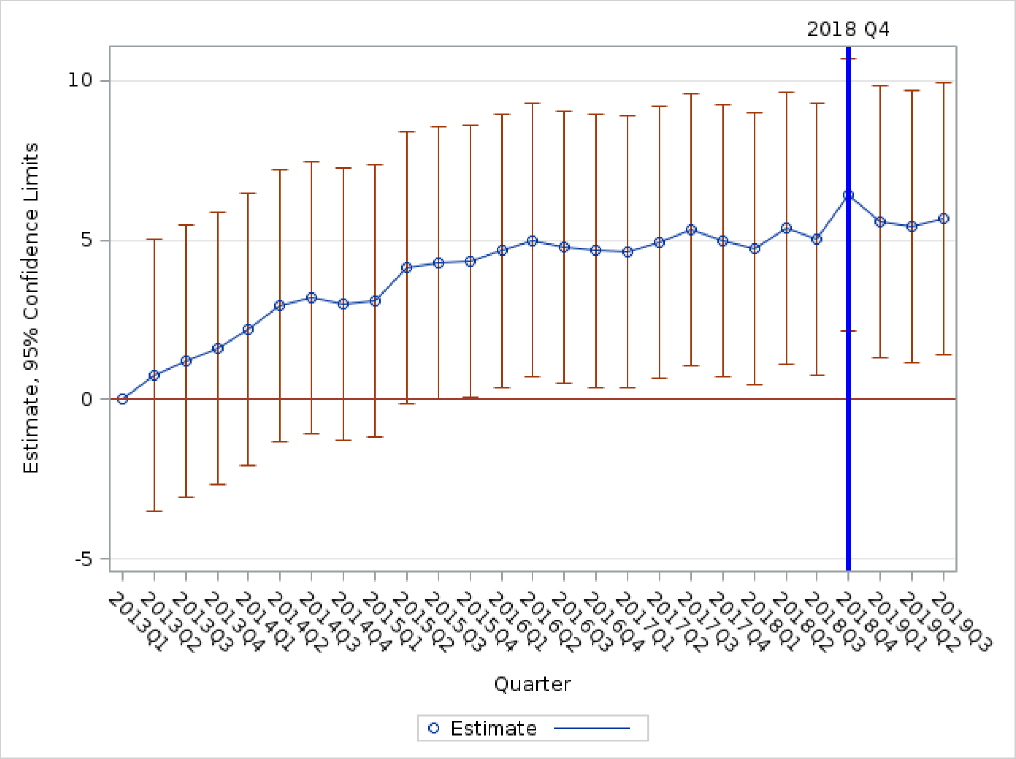

Inpatient surgery volume, by quarter

© 2025 Kilaru AS et al. *JAMA Health Forum*.

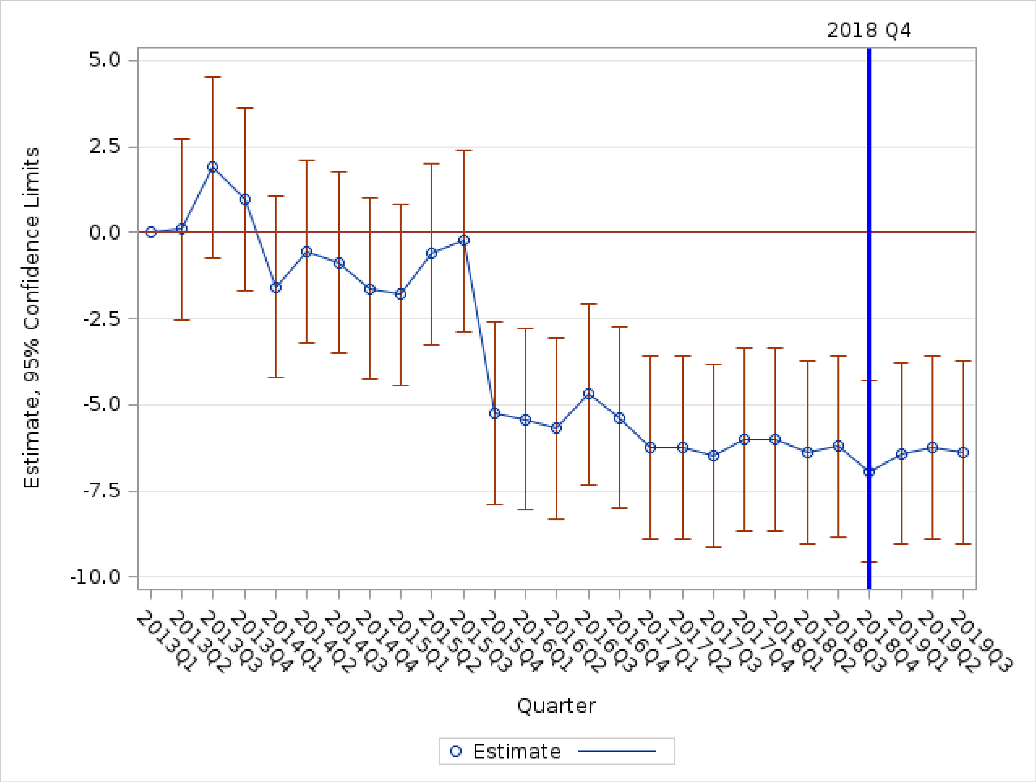

All surgery volume, by quarter

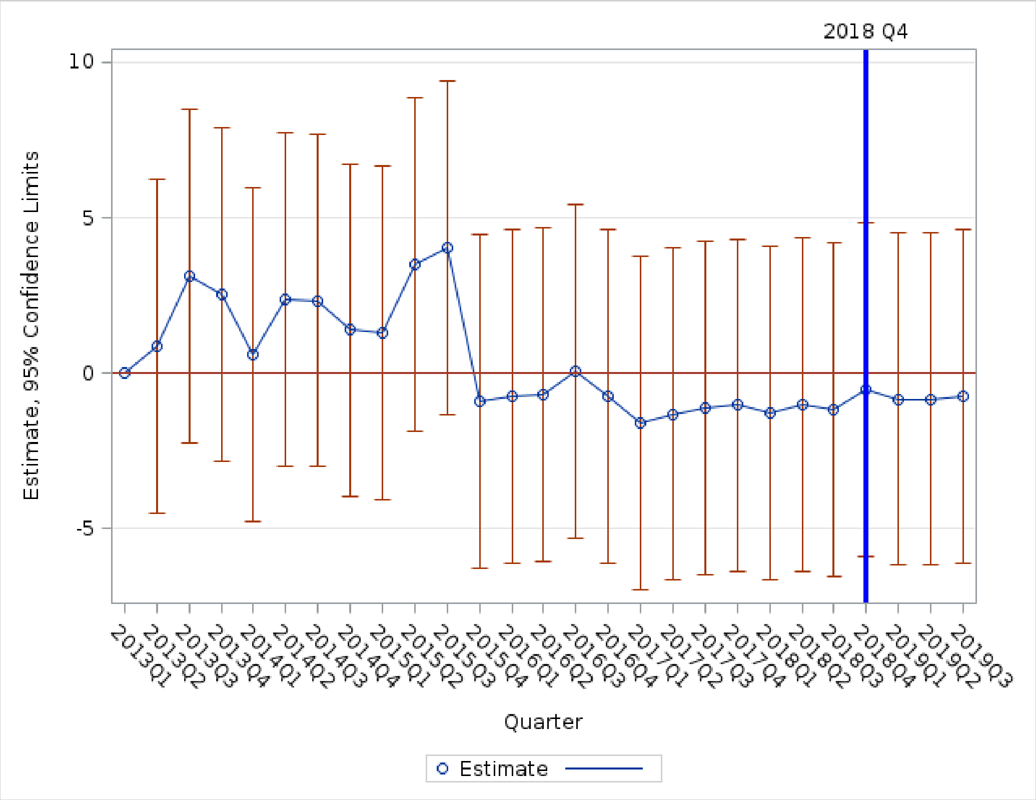

© 2025 Kilaru AS et al. *JAMA Health Forum*.
