# Supplementary material for: Savings Associated With Bundled Payments for Outpatient Spine Surgery Among Medicare Beneficiaries
Source: JAMA Health Forum. 2025 Jul 11;6(7):e251907. doi: 10.1001/jamahealthforum.2025.1907 (PMC12254892; doi:10.1001/jamahealthforum.2025.1907)
Supplement: Supplement 2. — Data Sharing Statement [file jamahealthforum-e251907-s002.pdf]

Data Sharing Statement

Kilaru. Savings Associated With Bundled Payments for Outpatient Spine Surgery Among Medicare Beneficiaries. *JAMA Health Forum*. Published July 11, 2025.
doi:10.1001/jamahealthforum.2025.1907

Data
Data available: No

Additional Information
Explanation for why data not available: This study uses Medicare data, which we are not allowed to share per the terms of our data use agreement. We would be happy to share our programming code and further details on methodology.
